# Fully Automated Purification of Small Molecules Using Online MicroSPE-UPLC-SPE

**DOI:** 10.1021/acs.analchem.6c00842

**Published:** 2026-09-17

**Authors:** Venkata Ganesh Jarubula, Gerd Grewing, Pauline Stark, Maximilian Frey, Dario Esposto, Simon Isfort, Marvin Hempel, Guillaume Patoine, Henriette Uthe, Alain Tissier, Gerd U. Balcke

**Affiliations:** † 28403 Leibniz Institute of Plant Biochemistry, Program Center MetaCom, Weinberg 3, Halle/S. D-06120, Germany; ‡ 587045 Axel Semrau GmbH, Stefansbecke 42, Sprockhövel D-45549, Germany; § Leibniz Institute of Plant Biochemistry, Department of Cell and Metabolic Biology, Weinberg 3, Halle/S. D-06120, Germany; ∥ Heidelberg University, Biochemistry Center, Heidelberg 69120, Germany

## Abstract

Structural annotation of metabolites remains a key challenge in metabolomics, as NMR-based structure elucidation for every unknown UPLC peak is limited by low sensitivity and time-consuming purification. To overcome these constraints, we developed an online microSPE-UPLC platform that enables fully automated, high-throughput peak fractionation. The system allows the injection of up to 1 mL of organic extract onto microSPE cartridges in a 96-well format for metabolite enrichment and cleanup. A dual-pump loading and refocusing strategy ensures quantitative transfer to UPLC, maintaining high chromatographic resolution while collecting narrow fractions on postcolumn microSPE. Fractions can be directly eluted with deuterated solvents for NMR analysis, eliminating drying steps and preserving volatile compounds. This approach enables milligram-scale isolation of natural products per compound within a single day. The workflow was validated using plant and fungal extracts, enabling the successful purification and characterization of several sesquiterpenoids and a diterpenoid.

## Graphical abstract

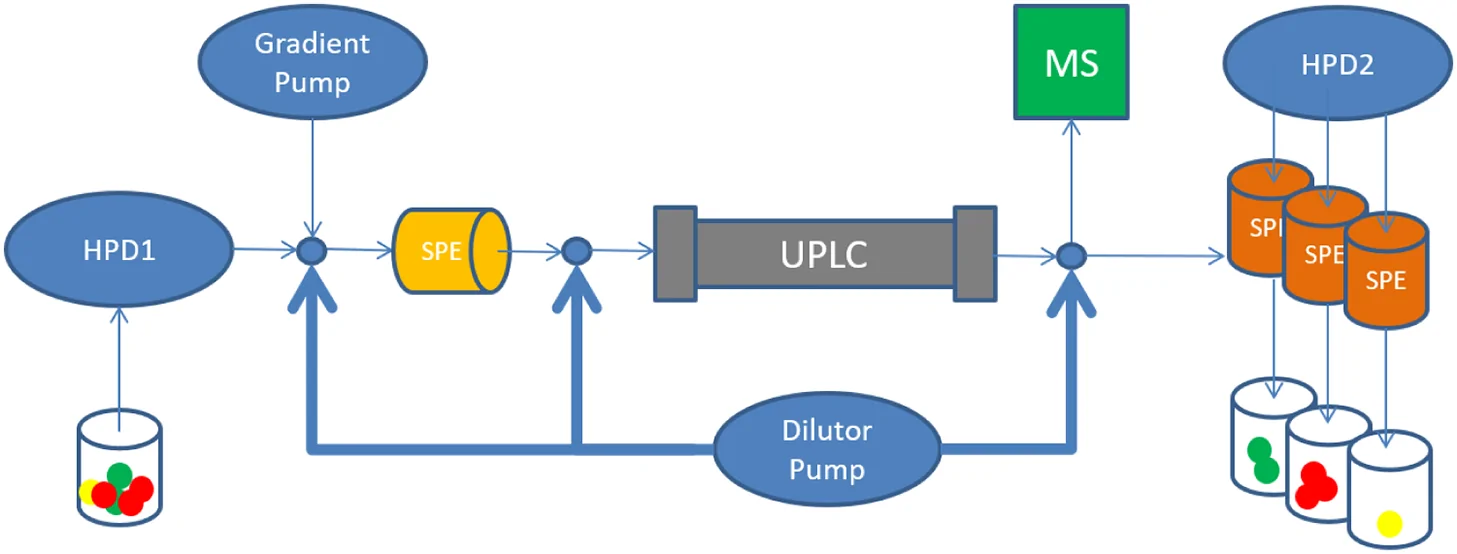

## Introduction

The purification of natural compounds is crucial for drug discovery, chemical biology and metabolomics, as these bioactive molecules serve as lead compounds for pharmaceuticals, agricultural agents, and other industrial applications. However, purifying compounds from complex natural sources using traditional methods such as thin-layer chromatography (TLC), solid-phase extraction (SPE), or preparative liquid chromatography (LC) can take weeks or even months. Moreover, these approaches often have limited chromatographic resolution, depend on chromophores, or require additional equipment for mass spectrometry compatibility. Additionally, preparative LC consumes large volumes of solvents, resulting in high costs.

Automated chromatographic systems for natural product isolation often rely on liquid fractionation, requiring re-extraction or extensive drying, which is especially critical for labile or volatile compounds.^1–2,3^ An important advance in terms of higher chromatographic performance is the integrated ultrahigh purification liquid chromatography (UPLC)-SPE system, which combines optimal peak separation with fraction collection to microSPE in microtiter plate format without the need for drying, enabling the purification and annotation of over 100 novel metabolites from *Medicago truncatula* extracts.^4^ However, the semipolar to apolar nature of the collected metabolites requires organic solvents for dissolution, limiting UPLC injection volumes to a few microliters. As a result, multiple recurrent fractionations are required to obtain sufficient material for downstream structural elucidation.

While large-volume enrichments to online SPE prior to reverse phase (RP)-UPLC exist, applications are limited to aqueous sample matrices like urine,^5,6^ human serum,^7,8^ plasma,^9^ saliva,^10^ groundwater or wastewater.^11,12,13,14^ In contrast, large volume injections of less polar analytes are limited to those samples dissolving in hydrophilic solvents (usually aqueous–organic solvent mixtures) unless organic extracts are manually loaded to SPE via dry load.^3^

Furthermore, in online SPE-LC coupling with reverse phase LC, the transfer of SPE-enriched compounds to the analytical column often leads to significant peak broadening, especially for polar and semipolar analytes. To alleviate this problem, the organic SPE effluent was repeatedly mixed with the aqueous mobile phase through multiple injection plugs, sequentially delivering desorbed analytes to the LC column head for peak refocusing.^15^ SPARK Holland introduced an SPE-UPLC system that mitigates peak broadening by continuously diluting organic SPE eluates with water during transfer from SPE to LC, ensuring that semipolar metabolites are trapped at the column head before separation.^16^ This system also employs high-pressure dispensers for injecting samples into 96-well microSPE cartridges.^17^ Nevertheless, automated large-volume injections of organic extracts onto RP-based SPE and separation by RP-UPLC are still not feasible, as they require immediate in situ sample dilution with water prior to SPE loading to capture semipolar natural compounds.^18^

To address these problems, we developed a novel automated online SPE-UPLC system featuring a dual-pump setup for sample loading, enrichment via 96-well microSPE cartridges, peak refocusing, and optional fractionation to individual SPE cartridges. This setup enables an injection volume up to 1000 μL of methanolic extract while preserving UPLC performance across a broad polarity range. It allows efficient purification of multiple semipolar analytes with ca. 90% recovery at ∼1 mg scale within 24 h. As proof of concept, we present an optimized workflow for enriching, purifying, and separating three sesquiterpenoids from *Bipolaris sorokiniana*.

## Experimental Section

### Biological Material

*Nicotiana benthamiana* leaves used as the material for recovery analysis were grown under a 18 h photoperiod at a constant temperature of 22 °C in a greenhouse of the Leibniz Institute of Plant Biochemistry (IPB Halle (Saale), Germany). Leaf material was snap-frozen and cryo-milled. 100 mg units of ground leaf matter was extracted using pure methanol (LC-MS, CHROMASOLV) to achieve a final concentration of 100 mg mL^–1^.

*Bipolaris sorokiniana* (ND90Pr, Bs) used in this study was obtained from the DSMZ, Braunschweig, Germany. It was cultured on modified CM medium with 1.5% agar under dark conditions at 28 °C for 21 days prior to inoculation.^19^ Spores were harvested and inoculated in YpSs medium (consisting of 4 g yeast extract, 15 g soluble starch, 1 g K_2_HPO_2_, 0.5 g MgSO_4_·7H_2_O, made up to 1000 mL) for further incubation of 5 days. Five liters of the fungal culture (OD600 = 4.8) was then extracted in two rounds using ethyl acetate (LC-MS, LiChrosolv). The final extract was dried using rotary evaporation and a TurboVap concentrator (MP Biomedicals) using a nitrogen gas stream and stored in −80 °C until use.

### Chemicals and Standards

The composition of a methanolic master mix containing eight compounds spanning a wide polarity range is provided in Figure 1. Pisiferic acid was purchased from Tokyo Chemical Industry Co., Ltd., Japan. All other compounds were purchased from Merck, Darmstadt, Germany. Methanolic stock solutions were combined to create an eight-compound mixture used for 5 μL UPLC injections (Table S1). A diluted version (5 μM per compound in 80% methanol, 20% water) was prepared for online SPE analyses. These concentrations fell within the linear MS-response range of the mass spectrometer.

**Figure 1:**
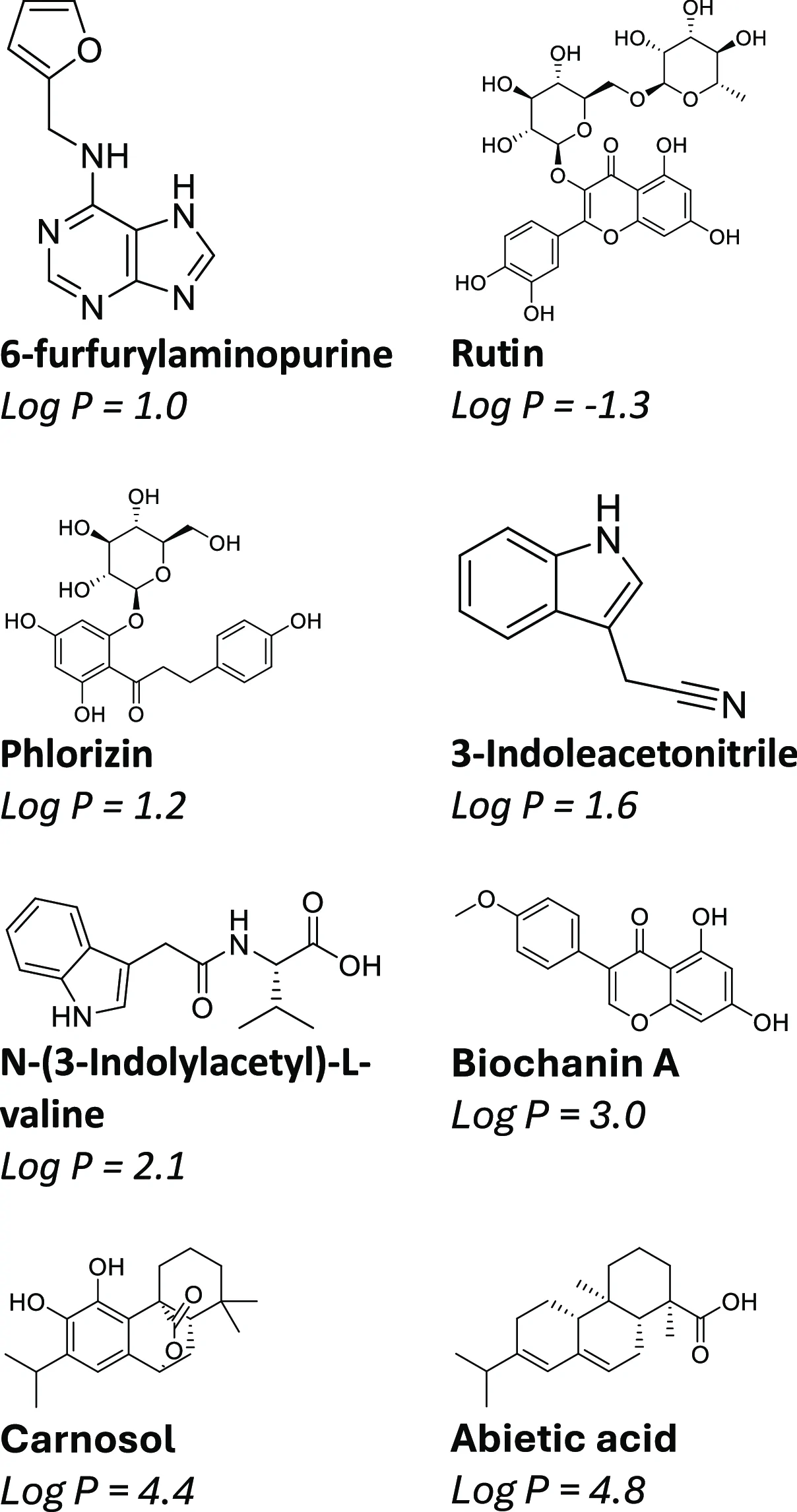
Eight natural products selected to develop the online-SPE-UPLC methodology; log P values represent the octanol–water coefficients predicted by ACD laboratories.

### Technical Equipment

#### Stationary Phases and UPLC Gradients

SPE was performed using a 96-well rack loaded with CHROspe cartridges packed with divinylbenzene (DVB) polymer (10 × 2 mm, 25–35 μm particle size, 10 mg fill weight), obtained from Spark Holland (Emmen, The Netherlands). Sorption of analytes from 80% methanolic solutions was achieved via in situ dilution with excess water upstream of the SPE cartridges. For direct desorption, compounds were eluted with 1 mL 100% methanol. For transfer to UPLC, analytes were eluted from SPE to the UPLC system with a 15 min isocratic flow of 20% aqueous 0.3 mM ammonium formate/formic acid (pH 3.2) mixed with 80% acetonitrile at a flow rate of 50 μL min^–1^ under aqueous dilution by 650 μL min^–1^. In case of abietic acid, also 100% acetonitrile was used for desorption. To prevent carryover, the transfer capillaries were then rinsed for 1 min with water at the same flow rate.

Eight standards were collected by online SPE-UPLC under acidic gradient conditions using a Nucleoshell RP18 column (100 × 4 mm, 2.7 μm) at 0.4 mL min^–1^ with a 1000 μL injection. Mobile phases consisted of 0.3 mM ammonium formate/formic acid, pH 3.2 (A1), and acetonitrile (B1). After 2 min 5% B1, a linear gradient ran from 5% to 95% B1 until 32 min, followed by re-equilibration to 37 min. The column temperature was maintained at 40 °C.

For quality control of collected fractions, metabolite separation was performed on a Nucleoshell RP18 (2 × 150 mm, particle size 2.7 μm, Macherey-Nagel) using a Waters ACQUITY UPLC System, equipped with a binary solvent manager and sample manager (20 μL sample loop, partial loop injection mode, 5 μL injection volume; Waters, Eschborn, Germany). Column flow rate and oven temperature were as above. The gradient started at 5% B1 for 0.75 min, increased linearly to 35% B1 until 1.5 min, increased linearly to 95% until 8 min, was held for 2 min, and then returned to initial conditions re-equilibrating until 12 min.

For high-resolution purification of sesquiterpenoids (SQTs) from *Bipolaris sorokiniana* extracts, we developed further chromatographic gradients. For acidic conditions, a Nucleoshell RP18 column (100 mm × 4 mm, 2.7 μm) was used and for neutral separations, a Nucleoshell PFP column (100 mm × 4 mm, 2.7 μm, Macherey & Nagel, Düren, Germany) was employed. Acidic separations involved 0.3 mM ammonium formate/formic acid (pH 3.2, A1) and acetonitrile (B1) with a column flow of 0.6 mL min^–1^ and 40 °C column temperature. Elution profile: 0–1 min, linear from 5% to 35% B1; 1–11 min, isocratic at 35% B1; 11.01–15 min, 35% A1 at 0.4 mL min^–1^; 15.01–21 min, linear from 35% to 95% B1 at 0.6 mL min^–1^; 21–24 min, 95% B1; 24.01–27 min, re-equilibration at 5% B1. For neutral conditions, we used 10 mM ammonium acetate (A2) with acetonitrile (B1) at a flow of 0.4 mL min^–1^: 0–4 min, linear from 5% to 45% B1; 4–20 min, 45% B1; 21–24 min, 95% B1; 24.01–26 min, re-equilibration to 5% B1.

Conventional UPLC was performed on a Waters ACQUITY system, while all other gradients were run on a SPARK platform (Figure 1).

#### Instrumentation for Purification

A prototype of a CHRONECT Symbiosis online-SPE-UPLC system was constructed in collaboration with Axel Semrau GmbH (Sprockhövel, Germany; Figure S1), which is equipped with the following hardware: a CHRONECT Robotic RTC PAL with Peltier Cooler [1], a CTC liquid and dilutor tool [2], and two injection ports [3], one fitted with a 5 μL loop for direct injection to LC and one with a 1000 μL loop for injection to SPE. For online SPE and fraction collection, a 96-well plate SPE unit (ACE) [4], two high- pressure dispensers (HPD) (both Spark Holland) [5], and two additional 6-port valves (VICI, Schenkon, Switzerland) [6] were integrated. Analyte trapping by SPE or UPLC, employed a dilutor pump (EPH30 UPLC dilution pump, Knauer GmbH, Berlin, Germany) [7] Chromatographic separations were performed using an SPH1299 binary gradient pump [8] and a Mistral column oven [9] (both Spark Holland). To reduce background concentrations of protic solvents during NMR-based structural analysis, the SPE cartridges can be automatically dried with nitrogen prior to elution with deuterated solvents. All liquid handling operations were controlled by the Chronos software version 5.3.9.0 (Axel Semrau GmbH, Sprockhövel, Germany

#### Mass Spectrometry

Analysis of small molecules was performed by QToF-MS/MS (ZenoToF7600, AB Sciex GmbH, Darmstadt, Germany) operating in negative and positive ion mode and controlled by SciexOS software (AB Sciex GmbH, Darmstadt, Germany). The source operation parameters were as follows: ion spray voltage, −4500 V (5500 V); nebulizing gas, 60 psi; source temperature, 600 °C; drying gas, 70 psi; curtain gas, 35 psi.

For MS^1^ measurements, precursor ion masses were scanned between 65 and 1250 Da with an accumulation time of 100 ms and a collision energy of −10 V for negative and 10 V for positive ionization. Selected ions for the eight-component master mix were quantified as [M-H] adducts throughout within a mass tolerance window of 5 ppm to the theoretical [M-H]. MS^2^-DDA-experiments were divided into 40 scan windows of 20 ms accumulation time fragmenting the most abundant MS^1^ signals. Throughout all MS/MS scans, a declustering potential of −35 V (35 V) was applied. Collision energies for all MS/MS were set to −35 V (35 V) and a collision energy spread of ± 25 V and elsewise to default settings. To avoid MS detector overloading, a flow-splitter right in front of the MS-inlet ensured a split ratio of 1 by 10 diverting a small fraction the UPLC effluent to the MS analysis. During fraction collection the LC effluent is completely directed to SPE collection.

#### Structure Elucidation, Quantification, and Purity Analysis by NMR

The isolated compounds were transferred into 5 mm NMR tubes (DEU-Quant, Deutero) within 600 μL CD_3_OD containing 0.01862 mM hexamethyldisiloxane (HMDSO, internal standard) purchased from Deutero GmbH (Kastellaun, Germany). ^1^H and 2D spectra (HSQC, HMBC, COSY, TOCSY, NOESY) were recorded on a Bruker Avance Neo 700 spectrometer operating at 700.344 MHz (13C: 176.122 MHz) using a 5 mm inverse detection cryoprobe (TCI). Quantitative ^1^H NMR spectra were recorded using the following parameters: digital resolution = 0.03 Hz/point, a pulse width = 8 μs (90°), relaxation delay = 25.0 s, acquisition time = 7.6 s, and number of scans = 128. All spectra were manually phase adjusted, automated baseline corrected, and proton and carbon chemical shifts were referenced to HMDSO (δ ^1^H = 0.062 ppm) and CD_3_OD (δ ^1^³C = 49.0 ppm), respectively. The software used for data acquisition was TopSpin version 4.4.1, and structure elucidation and integration were performed with MestReNova 15.0.1. For quantification, the peak area of the internal standard and three selected signals of the target compound were used (Table S5, Figure S28)

## Results and Discussion

### Design of Online MicroSPE-UPLC for Large Volume Injections and SPE Fraction Collection

We developed a prototype device for online microSPE-UPLC-SPE enabling automated, large-volume injections (up to 1 mL). All SPE steps are fully automated, including conditioning, loading, and online enrichment of target compounds using SPE cartridges in 96-well microtiter plate format, with peak refocusing during SPE eluate transfer to the UPLC column. A novel element, a dilution pump [1] was integrated to enhance the retention during sample loading (step 1) and transfer to UPLC (step 2) (Figure 2, Figure S1). It also dilutes the UPLC column effluent to enable trapping on individual microSPE cartridges for peak fractionation (Step 3).

**Figure 2:**
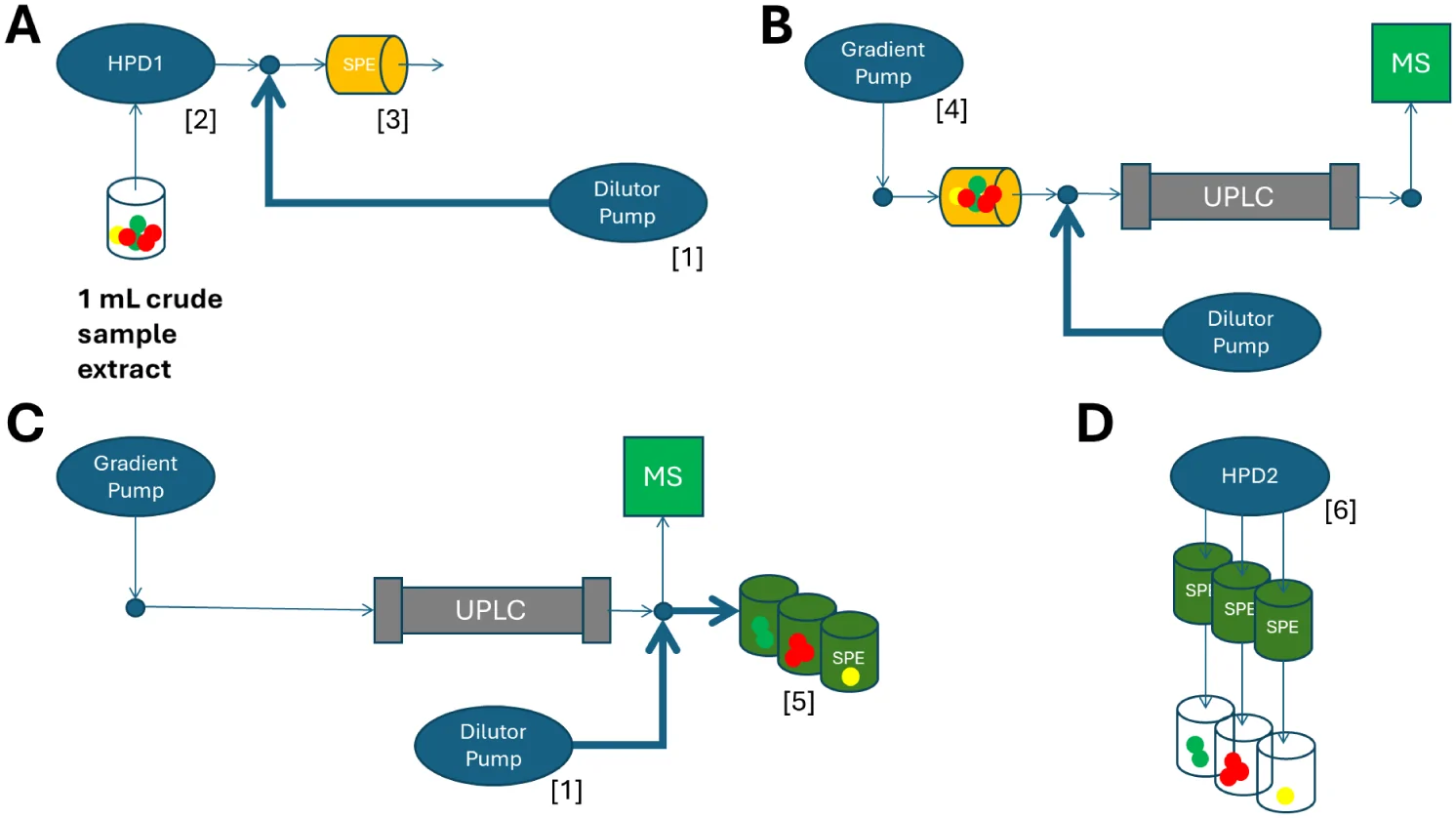
Four principal steps of online SPE-UPLC with SPE fraction collection. A. Loading to SPE with dilution, B. Transfer from SPE to UPLC with dilution (peak refocusing), C. UPLC separation with fraction collection to individual receiver SPE and dilution, D. Fraction desorption. An animation involving the flow pathways of each step can be found as Supporting Information Video S1.

Step 1: Compound enrichment by online SPE

All solvents for conditioning, washing, and loading of the SPE cartridges were applied using a high-pressure dispenser [2] (HPD; Spark Holland). Preconditioned microSPE cartridges were loaded with 80% methanolic crude extracts of *N. benthamiana* using the same HPD system. During sample loading (up to 1 mL), a dilution pump [1] introduced simultaneous dilution via a T-piece positioned right upstream of the microSPE inlet, which is clamped by a holder [3] (Figure 2, panel A). Thereby, the flow ratio between extract and dilution solvent can be adjusted freely. Advantageously, the crude plant extract can now be loaded directly from solutions of water-miscible organic solvents maintaining the solubility of semipolar standards.

Step 2: Transfer from SPE to UPLC

Sequential desorption of target analytes is achieved via the UPLC gradient pump [4] with real-time aqueous dilution of the SPE eluate to trap target analytes at the column head (Figure 2, panel B). This setup allows flexible adjustment of the elution-to-dilution ratio and the tailored elution gradients enable selective recovery of desired compounds, while hydrophobic matrix components like lipids and chlorophylls remain on the SPE cartridge and are subsequently flushed to waste.

Step 3: Chromatographic separation on UPLC with fraction collection to microSPE

Regular RP chromatography separates target analytes, which can be collected individually based on their elution time using one preconditioned microSPE cartridges [5] per compound (Figure 2, panel C). The UPLC dilution pump [1] is used to trap selected analytes on the collection SPE RP stationary phase.

Step 4: Elution from microSPE

If sufficient purity and quantity is reached in a single run, the cartridges [5] can be directly eluted, e.g., with suitable NMR-compatible deuterated solvents by a second HPD [6]. If more material is needed, repeated injections can be collected on the same cartridge. For analytes that require further purification, the cartridge [5] can repeat step 2 and 3 by performing an orthogonal 2D separation on a different stationary phase, pH or eluent. The entire 2D process can be fully automated (Figure S2)

### Method Optimization

First, we optimized a protocol for eight natural compounds spanning a wide polarity range providing a template method set for future developments, including all individual process steps (Figure 1, Table S1). The selected standards include amino groups (1,4,5; protonatable), carboxylic acids (5,8; deprotonatable), sugar moieties (2,3), aromatic or π-systems (1–8), heterocycles (1,2,4–7), and phenolic groups (2,3,6,7).

Step 1: Influence of the sample loading composition on the recovery

Using the HPD [2] and the dilution pump [1], final methanol–water mixtures ranging from 5% to 66% MeOH were adjusted to study solvent effects on recovery. Polar compounds (6-Furfurylaminopurine, Rutin, Phlorizin) were recovered efficiently only with low MeOH share upon 1000 μL SPE loading of 5 μM solutions of an eight-component standard mix (Figure 2), while semipolar and apolar substances were largely unaffected by solvent composition (Figure 3A). Thus, the ratio between dilutor pump and methanolic sample extracts should be adjusted to 10% methanol or lower to completely trap also hydrophilic compounds.

**Figure 3:**
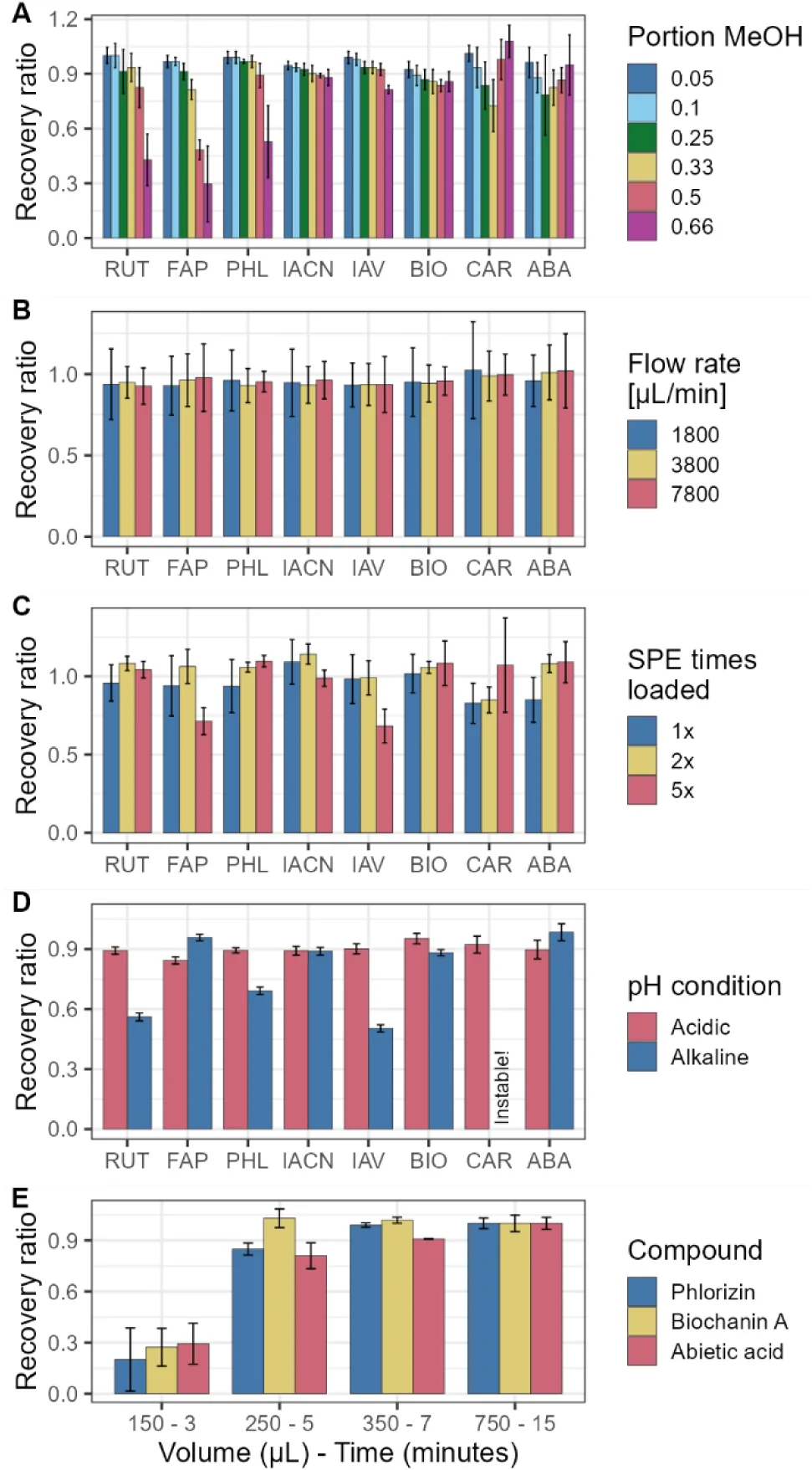
Method parameters influencing the recovery during online-SPE-UPLC: A) Impact of varying loading buffer methanol percentages, B) Influence of sample loading speed, C) Influence of repeated SPE cartridge loading, D) Influence of the loading pH E) Influence of the desorption volume used during the transfer step. Error bars indicate standard deviations derived from error propagation (*n* = 3). The above abbreviations with their metabolite names are described as follows, rutin (RUT), 6-furfurylaminopurine (FAP), phlorizin (PHL), 3-indolylacetonitrile (IACN), N-(3-indolylacetyl)-l-valine (IAV), biochanin A (BIO), carnosol (CAR), and abietic acid (ABA).

Step 1: Influence of sample loading speed on the recovery

Next, we examined the loading flow rate and solvent composition during sample loading onto the DVB microSPE cartridge (Figure 1, panel A). A 1000 μL methanolic solution of eight standards at 5 μM each was loaded onto the SPE [3] via HPD [2] at 200 μL min^–1^. The dilutor pump [1] delivered 1800, 3800, and 7800 μL min^–1^, resulting in 10%, 5%, and 2.5% of MeOH as the final share, respectively. After bypassing steps 2–3, desorption was performed with 1 mL of MeOH. Recovery was assessed by regular UPLC-MS comparing control and SPE-processed samples (Figure 3B). Since recovery for all analytes was higher than 93% and remained consistent across flow rates, a flow rate of 3800 μL min^–1^ was used for subsequent experiments, which results in a 5 min phase to load the sample.

Step 1: Influence of the sample matrix on the recovery

To assess potential capacity limitations of the SPE cartridge with concentrated biological extracts, a range-finding test was performed to determine the maximum loading capacity of the SPE microcartridge. Methanolic *Nicotiana benthamiana* leaf extracts (100 mg mL^–1^ in 80% MeOH) were spiked with eight standards (5 μM each). 1 mL portions of this solution were loaded onto the same microSPE cartridge either once, two times, or five times, followed by methanolic desorption (1 mL) and recovery analysis was carried out against untreated controls of identical extract concentration.

Repeated loading of plant extracts showed consistently high recoveries after five uses of the same DVB microSPE cartridge, except for 6-furfurylaminopurine (71%) and N-(3-indolylacetyl)-l-valine (68%), indicating a slightly limited capacity for very hydrophilic or amino-functionalized metabolites. Overall, the DVB sorbent demonstrated excellent reusability and high loading capacity (Figure 3C).

Step 1: Influence of loading buffer pH on compound recovery

Metabolites often contain carboxylic, hydroxy, or amino groups that can be protonated or deprotonated depending on the pH, thereby altering their charge, polarity, and retention on RP sorbents. To examine this effect, eight natural compounds (5 μM each) were loaded onto SPE cartridges from 80% methanolic buffers adjusted either to pH 2.5 with formic acid or to pH 10.5 with ammonia.

As shown in Figure 3D, semi- to midpolar compounds bearing phenolic or carboxylic groups (rutin, phlorizin, N-(3-indolylacetyl)-l-valine) exhibited reduced retention on DVB microSPE at high pH, whereas the addition of formic acid decreased the recovery of the hydrophilic, amino-functionalized compound 6-furfurylaminopurine. In contrast, hydrophobic compounds (biochanin A, abietic acid) were unaffected by loading pH, regardless of their functional groups.

Step 2: Determination of the transfer volume

After SPE loading, analytes are transferred to the UPLC and trapped at the column head before gradient elution (Figure 2, panel B). A low flow using organic solvents promotes SPE desorption, while an excessive water flow dilutes the eluent to enable peak refocusing at the column head. During the transfer phase (tandem configuration, Figure 2B), pressure is limited to below 300 bar due to the plastic-housed cartridges; however, no such limitation applies during the other steps of the procedure. To minimize the system pressure, we employed 4 mm inner diameter core–shell UPLC columns, which maintain backpressures below 250 bar even at a total flow of 700 μL min^–1^ comprising 650 μL min^–1^ of water from the dilutor pump [2] and 50 μL min^–1^ from the gradient pump [4] (Figure 2B).

By adjusting the desorption volume at the gradient pump, we identified the minimum volume of 750 μL of organic solvent required for complete elution of all eight compounds from the DVB SPE, effectively reducing the transfer time to only 15 min for semipolar compounds such as phlorizin and biochanin A (Figure 3E). We used 80% acetonitrile for these analytes, but needed 100% acetonitrile to fully transfer abietic acid. Even with the higher organic content, hydrophobic compounds still required larger desorption volumes for full elution, resulting in longer processing times.

Steps 1–3: Recovery of eight compounds using SPE-UPLC with collection to SPE

With steps 1 and 2 being optimized, we injected 1000 μL of 80% methanolic solutions of the eight natural compounds at 5 μM each and combined steps 1–3 with collection of individual fractions on receiver SPE. After desorption of SPE-collected fractions by 1 mL MeOH, the recoveries were compared against nontreated controls using regular UPLC with 5 μL injections.

Because the polarity range between the most hydrophilic (rutin) and most hydrophobic (abietic acid) compounds spans a wide range of octanol–water coefficients (Figure 1), no single method could quantitatively recover all analytes under one set of conditions. Although six of the eight compounds showed high recovery using 80% acetonitrile (>93% recovery) using 750 μL transfer volume, rutin and abietic acid showed reduced recovery (Table 1). Hydrophobic compounds require 100% acetonitrile during the SPE-to-UPLC transfer (step 2) to improve recovery (as shown for abietic acid), but under these conditions hydrophilic compounds were insufficiently retained at the UPLC column head. Thus, lowering the organic content in step 2 will further improve the recovery of hydrophilic analytes such as rutin but is not demonstrated here.

**Table 1:** Recovery of Eight Natural Compounds Purified After 1 mL Injection to Online SPE-UPLC and Fraction Collection by MicroSPE^a^

	% acetonitrile used for desorption	% recovery and intraday precision (standard deviation, *n* = 3)	% recovery and interday precision (standard deviation, *n* = 9)
Rutin (RUT)	80	69 ± 9	70 ± 9
6-Furfurylaminopurine (FUR)	80	99 ± 3	98 ± 4
Phlorizin (PHL)	80	103 ± 7	101 ± 9
N-(2-Indolylacetyl)-valine (IACN)	80	93 ± 8	91 ± 8
3-Indolylacertonitrile (IAV)	80	97 ± 4	97 ± 6
Biochanin A (BIO)	80	95 ± 2	96 ± 6
Carnosol (CAR)	80	98 ± 3	95 ± 7
Abietic acid 80% (ABA)	80	5 ± n.d.	n.d.
Abietic acid 100% (ABA)	100	94 ± 6	94 ± 7

a Standard deviations derived from error propagation (N = 3).

The experiment was repeated on three individual days showing results comparable interday precision (Table 1). Together, we provide a validated, reproducible method with a median of 97% recovery rate for natural compounds. Optimized online SPE-UPLC parameters for fraction collection are listed in Table 2 and can serve as a template for future purifications. They were validated using eight natural products with diverse polarity and pH-responsive functional groups (Figure 1).

**Table 2:** Summary of Optimized Parameters for Fully Automated 2D Peak Purification by Online-SPE-UPLC-SPE^a^

				**loading**				**sample transfer/peak refocusing**						**UPLC**		
Step	Meaning	how done	stationary phase	injection volume	sample loading	sample solvent	dilution rate	dilution solvent	desorption rate	desorption solvent	dilution rate	dilution solvent	desorption/refocusing time	solvent A	solvent B	fraction collection
				[μL]	[μL min^–1^]		[μL min^–1^]		**[μL** min^–**1**^]		**[μL min** ^**–1**^]		**[min]**			
1	cartridge conditioning	1 mL THF, 1 mL MeCN, 1 mL 1%HOAc, 1 mL H2O	DVB	-	-	-	-	-	-	-	-	-	-	-	-	-
2	1^st^ purification: RT check	acidic gradient	RP18	100–1000	200	80% MeOH	3800	H_2_O	50	MeCN	650	H_2_O	15	0.3 mM ammonium formate/formic acid (pH3.2)	MeCN	no
3	1^st^ purification: fraction collection	acidic gradient	RP18	100–1000	200	80% MeOH	3800	H_2_O	50	MeCN	650	H_2_O	15	0.3 mM ammonium formate/formic acid (pH3.2)	MeCN	yes
4	2^nd^ purification: RT check	neutral gradient	PFP	no loading, only desorption from SPE (step 3)					50	MeCN	650	H_2_O	15	10 mM ammonium acetate (pH6.7)	MeCN	no
5	2^nd^ purification: fraction collection	neutral gradient	PFP	no loading, only desorption from SPE (step 3)					50	MeCN	650	H_2_O	15	10 mM ammonium acetate (pH6.7)	MeCN	yes
6	fraction elution from SPE	1 mL 80% MeCN/1 mL MeCN*	DVB	-	-	-	-	-	-	-	-	-	-	-	-	-

a Abbreviations: THF – tetrahydrofurane, MeCN – acetonitrile, HOAc – acetic acid; *hydrophobic compounds require 100% organic solvents such as MeCN or 2-propanol to fully elute DVB-SPE cartridge material.

### Chromatographic Performance

Comparing 5 and 500 μL injections of a 5 μM compound mixture without fraction collection demonstrated superior chromatographic performance of online-SPE-UPLC over conventional UPLC, with substantially higher signal intensities (Figure 4). Blank injections following 500 μL large-volume injections showed minimal carryover (Figure S3).

**Figure 4:**
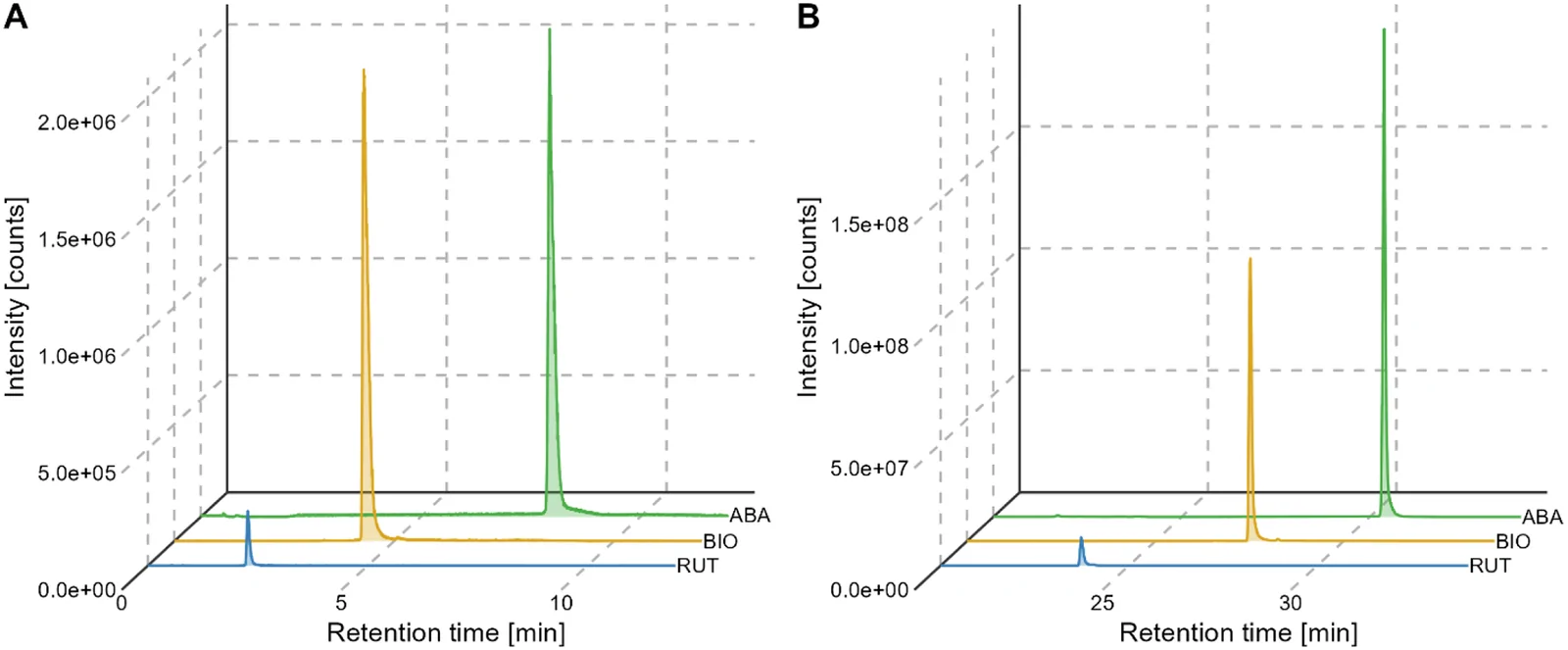
Chromatographic performance of regular UPLC (5 μL injection, panel A) as compared to online-SPE-UPLC (500 μL injection, panel B) with optimized parameter settings for steps 1–2 omitting any peak fractionation. Comparison peak width at half height [s] UPLC/online SPE-UPLC: Rutin (4.0/5.5); Biochanin A (8.8/4.8); Abietic acid (7.8/4.0).

### Purification of Pisiferic Acid and Three Sesquiterpenes from a Fungal Extract

With steps 1–3 being optimized, we proceeded to purify three sesquiterpenes (SQT) from an 80% methanolic extract of the plant-pathogenic fungus *Bipolaris sorokiniana* obtained from a dried culture pellet (Figure S4). As an internal standard, 215 μM pisiferic acid was spiked to the same extract and we injected 15 times 400 μL of this extract onto individual DVB SPE cartridges and desorbed to a RP18 UPLC column with peak-refocusing via water-dilution prior to chromatographic separation (Figure S5). Using a 27 min acidic separation gradient we first fractionated the four compounds to four individual SPE receiver cartridges. This was followed by a second purification by a neutral gradient on a pentafluorophenyl (PFP) stationary phase (26 min run time) with direct desorption of the collected fractions from the receiver SPE to the UPLC column head (Figure S2).

A comparative MS signal analysis of the crude extract yielded recovery rates of 89.5% for SQT1, 86.4% for SQT2, 3% for SQT3, and 86.4% for pisiferic acid; although matrix effects were not specifically investigated, the resulting signal intensities fell within the linear calibration range. The two-step automated purification yielded 125 μg SQT1, 574 μg SQT2, 32 μg SQT3, and 259 μg pisiferic acid (64% based on quantitative NMR). Compound identification by NMR and MS/MS assigned SQT1 (Table S2, Figures S6–S12) as helminthosporic acid and SQT2 (Table S3, Figures S13–S19) as helminthosporal acid, both compounds demonstrated high purity (Figure S11, S18). Whereas Figure S20 shows that the pH value during the consecutive UPLC strongly affects the retention time of organic acids, enabling improved purification.

SQT3 was determined as prehelminthosporol, a sesquiterpene lactone with the formula C_15_H_22_O_2_, which is consistent with a new peak emerging at 11.7 min at [M + H] 235.170 Da during quality control after NMR on a separate UPLC (Figure S26). The collected original compound ([M-H] = 251.165, 11.0 min), which was still present in the raw extract, degraded during the drying step needed to remeasure NMR spectra, which was associated with a signal increase of [M + H] = 235.170 Da as measured after NMR structure elucidation. This is consistent with a lactonization between C1 and C16 during the drying process prior to NMR and would also explain the low recovery rate of the original SQT3 itself (Table S4, Figure S21–S27).

The three compounds belong to a group of seco-sativene sesquiterpenoids produced by the Ascomycete *Bipolaris sorokiniana* (telemorph: *Cochliobilus sativus*).^20^ The stereochemistry of helminthosporic acid (SQT1) was previously established by X-ray diffraction.^21^ The three compounds belong to a group of seco-sativene sesquiterpenoids produced by the Ascomycete *Bipolaris sorokiniana* (telemorph: *Cochliobilus sativus*).^20^ The stereochemistry of helminthosporic acid (SQT1) was previously established by X-ray diffraction.^21^ For SQT1-SQT3, our spectral results match well with NMR and MS data of the respective first isolations^21,22,23^ (Table S5, Figure S28).

## Conclusions

In summary, we developed a fully automated online microSPE-UPLC device and a modular workflow to rapidly purify multiple compounds from a single methanolic extract. Organic extracts of up to 1000 μL can be injected into the UPLC via microSPE cartridges by simultaneously diluting injections with water during sample loading, thereby maintaining excellent chromatographic separation performance. MS-based recovery analysis of natural compounds covering a wide polarity range allowed nearly quantitative enrichment and isolation for structural elucidation. When the purification was repeated using orthogonal chromatographic approaches, recoveries still approached 90% (based on MS-analysis). This recovery could be further improved by widening collection windows and using alternative stationary phase chemistry. While strongly hydrophilic or amino group-functionalized compounds would require further method optimization, including alternative trapping strategies prior to chromatography, our method efficiently isolates analytes with a wide polarity range (with log P values ranging from −1.4 to 4.8) from concentrated sample matrices (100 mg FW mL^–1^). Our method development showed that up to 5 mL of crude plant extract can be processed by UPLC without significant losses and carryover, demonstrating the high loading capacity of DVB microSPE cartridges while maintaining good UPLC performance. In our experience, the DVB SPE cartridges can be used more often than five injections, but lifetime metrics were not investigated in greater detail. Traditional preparative HPLC can process samples in gram scale, but it typically requires high flow rates (10–200 mL per min^–1^) and large amounts of solvents per run.^24,25^ Traditional fractionation methods also depend on liquid fraction collections, which involve evaporation steps and rebuffering, which can lead to loss or degradation of sensitive or volatile compounds.^26,27^ Although coupling offline solid phase extraction (SPE) to analytical UPLC does reduce the solvent requirements, it still involves numerous hands-on sample handling steps and can take time anywhere from several days to a few weeks to complete.^26,28^ The UPLC-SPE-NMR platform described by Bhatia et al. is the closest comparison in published research so far, having identified over 100 metabolites from *Medicago truncatula*. However, their system can only handle injection volumes of a few microliters.^4,29,30^

Our system addresses this limitation by allowing much larger sample injections up to 1000 μL (μL) of methanolic extract, thanks to simultaneous dilution with water during the SPE loading step. This represents roughly a 100 to 200-fold increase in injection volume compared to previous platforms and assures best possible solvation of less polar compounds before injection. While the SPARK Holland SPE-UPLC system also uses online dilution, it has not been shown to work with large volumes of organic extracts or to collect fractions in a way that would allow for high-purity, NMR-ready samples.^31^

To our knowledge, there are no prior examples in the online SPE-LC literature of a fully automated, two-dimensional orthogonal purification workflow that combines direct elution into deuterated solvent for NMR, as we have demonstrated here. Our method uses only about 30 mL of solvent per run, dramatically reducing both costs and waste by up to 100 folds compared to conventional preparative HPLC. Additionally, our DVB microSPE cartridges can be reused for repeated loading after conditioning, thereby improving cost efficiency even further. Table S6 highlights the major advantages of our approach in throughput, recovery, and sustainability compared to traditional and current automated systems.

Compared to traditional methods like TLC or preparative HPLC, our approach offers lower capacity but superior UPLC-based separation performance, essential for complex matrices and eliminates manual sample handling during analyte separation and collection. Much lower solvent consumption as compared to preparative separation methods and the reusability of SPE cartridges make the online SPE-UPLC-SPE approach more cost-effective. Moreover, by eliminating liquid fraction collection, the method is also suitable for volatile compounds.

It is also important to note that the current system still relies on plastic-housed microSPE cartridges. These cartridges sustain pressures up to 300 bar limiting the transfer step, where sorbed solutes are transferred to the analytical column (Figure 2B). Because pressures from both columns add up in the tandem configuration, the method employs core–shell technology and 4 mm ID columns instead of conventional UPLC columns to reduce system pressure while increasing loading capacity. Alternatively, at the expense of increased transfer time and decreased chromatographic performance, lower flow rates could be used together with 2 mm ID UPLC formats or classic HPLC columns. These parameters are easily scalable for laboratories using different configurations.

Although the SPE-UPLC method requires time for sample loading and transfer to the UPLC column, potentially limiting throughput, parallelization of SPE conditioning and loading of subsequent samples during the UPLC could minimize the time per injection, reducing the effective processing time to 30 min per run. Thus, with similarly high recovery rates, an organic extract containing a compound at 40 μg mL^–1^ could be injected ∼50 times within just over a day using a 2D purification approach (25 injections in 1D and 25 in 2D).

To demonstrate this capability, 100–600 μg of high-purity compounds were isolated from a single sample matrix within 24 h, allowing sequential structural elucidation of three sesquiterpenes. The yield of the most abundant compound remained below 1 mg due to limited productivity of the fungal strain, not due to instrumental constraints. Thus, with similarly high recovery rates, an organic extract at 40 μg mL^–1^ could be injected ∼50 times within just over a day using a 2D purification approach (25 injections in 1D and 25 in 2D).

Combining fraction collection with UPLC poses challenges for rapid fraction collection. Typical collection windows range from 20 to 100 s and provide varying purification efficiency depending on sample complexity. In our experience, two-dimensional chromatographic separation is generally required to achieve sufficient purity of natural extracts for NMR analysis. We found that combining pH changes during UPLC with changes in stationary-phase chemistry had the greatest impact on separation performance.

The full equipment is commercially available via Axel Semrau GmbH, Sprockhövel, Germany.

## Supplementary Material





## Notes

Chemical properties of the test compounds used in this study; Detailed illustration describing the instrumental setup; Method validation of the online microSPE-UPLC-SPE workflow and purification performance; automated purification and characterization of fungal sesquiterpenes: representative chromatograms, NMR, HR-MS/MS, and purity analyses confirming efficient isolation and compound identity; method performance: Chromatographic behavior, ionization, and transformation studies demonstrating workflow robustness (PDF)
Video animation illustrating the individual steps of online microSPE-UPLC with large volume injection and peak fractionation to individual microSPE cartridges (MP4)
