# Supplementary material for: Fully Automated Purification of Small Molecules Using Online MicroSPE-UPLC-SPE
Source: Anal Chem. 2026 Sep 17;98(38):27885–94. doi: 10.1021/acs.analchem.6c00842 (PMC13629167; doi:10.1021/acs.analchem.6c00842)
Supplement: Supplementary file 1 [file ac6c00842_si_001.pdf]

Supporting Information

Fully Automated Purification of Small Molecules Using Online-microSPE-UPLC-SPE

Venkata Ganesh Jarubula[a], Gerd Grewing[b], Pauline Stark[a], Maximilian Frey[c,d], Dario Esposto[c], Simon Isfort[c], Marvin Hempel[c], Guillaume Patoine[a], Henriette Uthe[a], Alain Tissier[c] and Gerd U. Balcke[a*]

[a]Leibniz Institute of Plant Biochemistry, Program Center MetaCom, Weinberg 3, D-06120 Halle/S., Germany,

[b]Axel Semrau GmbH, Stefansbecke 42, D-45549 Sprockhövel, Germany,

[c]Leibniz Institute of Plant Biochemistry, Department of Cell and Metabolic Biology, Weinberg 3, D-06120 Halle/S., Germany,

[d]Heidelberg University, Biochemistry Center, 69120 Heidelberg, Germany

*Email: gerd.balcke@ipb-halle.de

Supporting Information: Table of Contents

Table S1	**Chemical properties of the test compounds used in this study** (Results and Discussion, Method Optimization)
Fig. S1-S2	**Detailed illustration describing the instrumental setup** (Experimental Section, Technical Equipment; Results and Discussion, Design of online micro-SPE-UHPLC for large volume injections and SPE fraction collection)
Fig. S3	**Method validation of the online micro-SPE-UHPLC-SPE workflow and purification performance** (Results and Discussion, Method Optimization)
Fig. S4-S24<br>Table S2-S5	**Automated purification and characterization of fungal sesquiterpenes: Representative chromatograms, NMR, HR-MS/MS, and purity analyses confirming efficient isolation and compound identity** (Results and Discussion, Purification of pisiferic acid and three sesquiterpenes from a fungal extract)
Fig. S3, S11, S18, S20, Table S6	**Method performance: Chromatographic behavior, ionization, and transformation studies demonstrating workflow robustness** (Results and Discussion, Method Optimization)
Video S1 Flow path online SPE-FC	**Video animation illustrates the individual steps of online microSPE-UPLC with large volume injection and peak fractionation to individual microSPE cartridges** (provided as a separate file: Video S1 Flow path online SPE-FC.mp4)

*Email: gerd.balcke@ipb-halle.de

Table S1. Detected adduct, retention time (control measurements using regular UHPLC) and SMILES notation of the eight standard compounds used in this study.

S.No	Compound Name	Concentration (µM)	$(M\text{-}H)^-$ (m/z)	Retention time	Smiles
1.	6-Furfurylaminopurine	10	214.07	5.4	*C1=COC(=C1)CNC2=NC=NC3=C2NC=N3*
2.	Rutin	10	609.14	5.7	*C[C@H]1[C@@H]([C@H]([C@H]([C@@H](O1)OC[C@@H]2[C@H]([C@@H]([C@H]([C@@H](O2)OC3=C(OC4=CC(=CC(=C4C3=O)O)O)C5=CC(=C(C=C5)O)O)O)O)O)O)O*
3.	Phlorizin	10	435.13	6.77	*C1=CC(=CC=C1CCC(=O)C2=C(C=C(C=C2O[C@H]3[C@@H]([C@H]([C@@H]([C@H](O3)CO)O)O)O)O)O)O*
4.	3-Indolylacetonitrile	50	155.06	8.4	*C1=CC=C2C(=C1)C(=CN2)CC#N*
5.	N-(3-Indolylacetyl)-L-valine	10	273.12	7.4	*CC(C)[C@@H](C(=O)O)NC(=O)CC1=CNC2=CC=CC=C21*
6.	Biochanin A	10	283.06	10.9	*COC1=CC=C(C=C1)C2=COC3=CC(=CC(=C3C2=O)O)O*
7.	Carnosol	6	329.17	12.95	*CC(C)C1=C(C(=C2C(=C1)[C@@H]3C[C@@H]4[C@@]2(CCCC4(C)C)C(=O)O3)O)O*
8.	Abietic acid	1000	301.21	17.1	*CC(C)C1=CC2=CC[C@@H]3[C@@]([C@H]2CC1)(CCC[C@@]3(C)C(=O)O)C*

*Email: gerd.balcke@ipb-halle.de

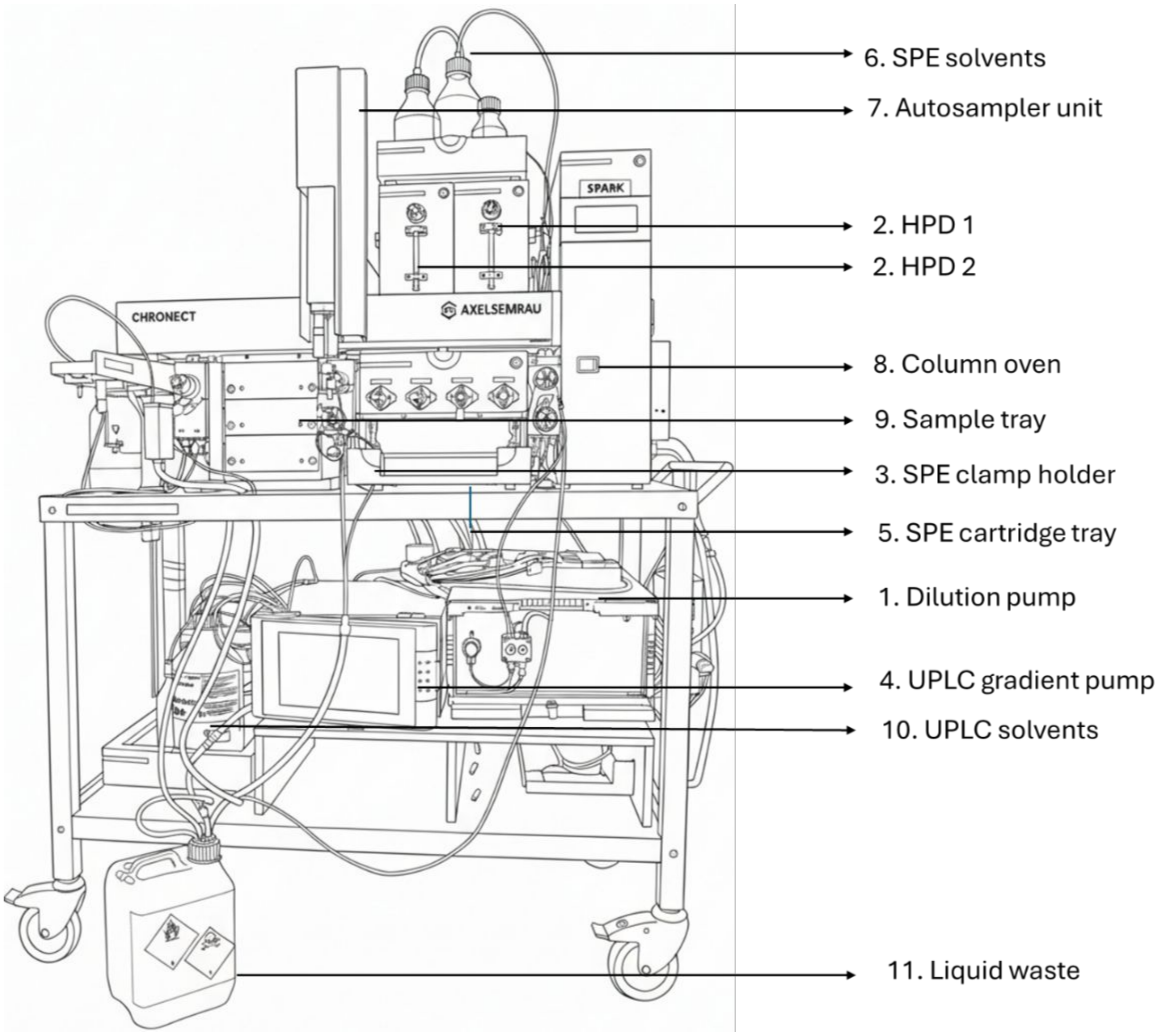

Figure S1. The instrument shown is an integrated CHRONECT Symbiosis system designed for online solid-phase extraction (SPE) coupled to ultra-performance liquid chromatography (UPLC). It consists of an Autosampler unit (7) that handles sample injection via the SPE clamp holder (3) and SPE cartridge tray (5). Sample processing is driven by the Dilution pump (1) and two High-Pressure Dispensers (HPD 1 & HPD 2) (2), which control the flow of SPE solvents (6) for enrichment and conditioning. Separated metabolites are run through the column located in the Column oven (8), with separation being performed by the UPLC gradient pump (4) using UPLC solvents (10), while the Sample tray (9) holds fractions or standards. All solvent and system waste is collected in the Liquid waste (11) container. *Original photograph by the author, modified into a graphical illustration using Google Gemini (Free Version, 2025; google.com)*

*Email: gerd.balcke@ipb-halle.de

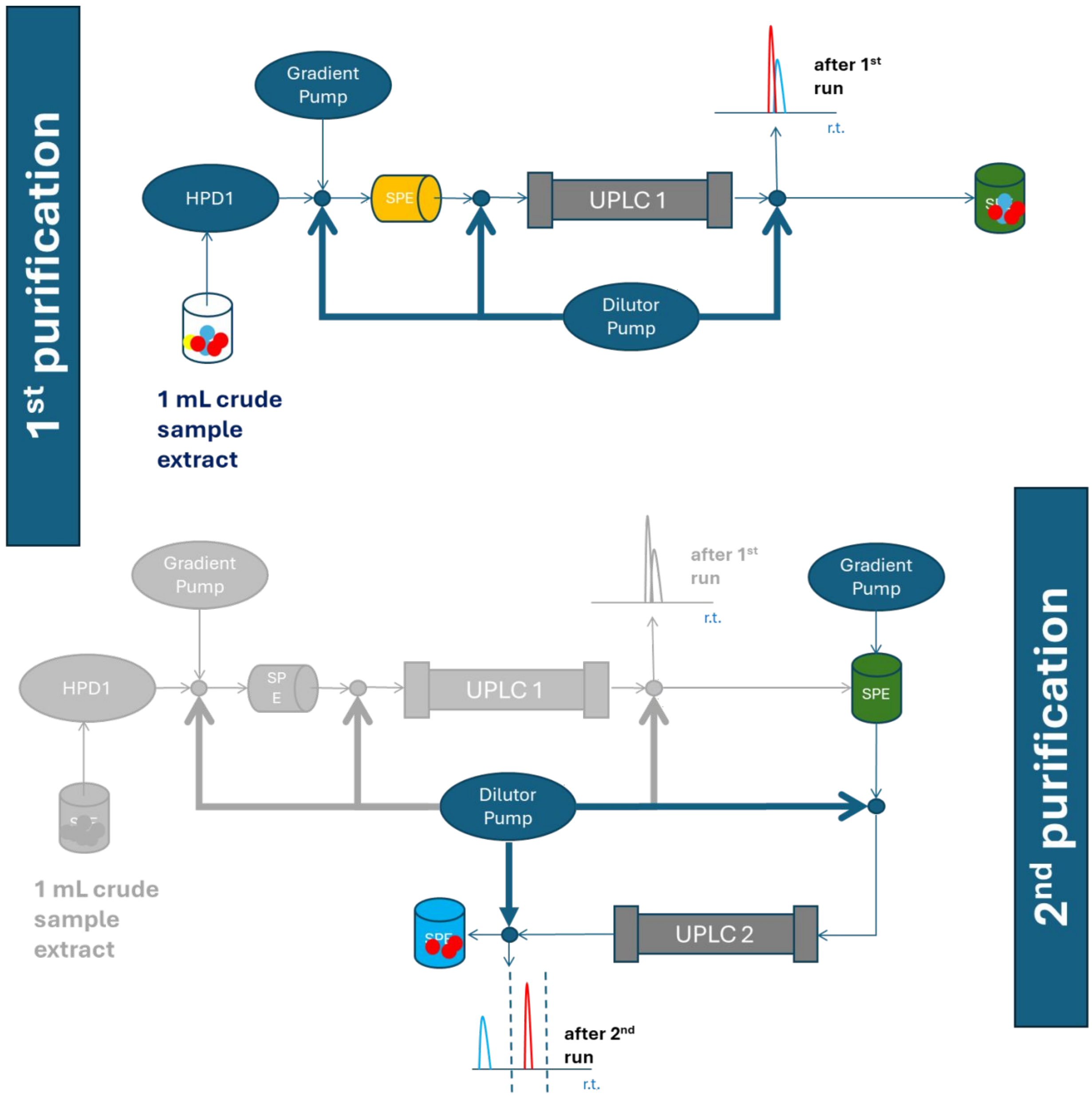

Figure S2. 2D-purification with consecutive peak separations. E.g. 1. Purification on RP18 using an acidic gradient an acetonitrile and fraction collection to a receiver SPE, 2. Desorption from the receiver SPE and further purification on PFP using a neutral gradient and methanol.

*Email: gerd.balcke@ipb-halle.de

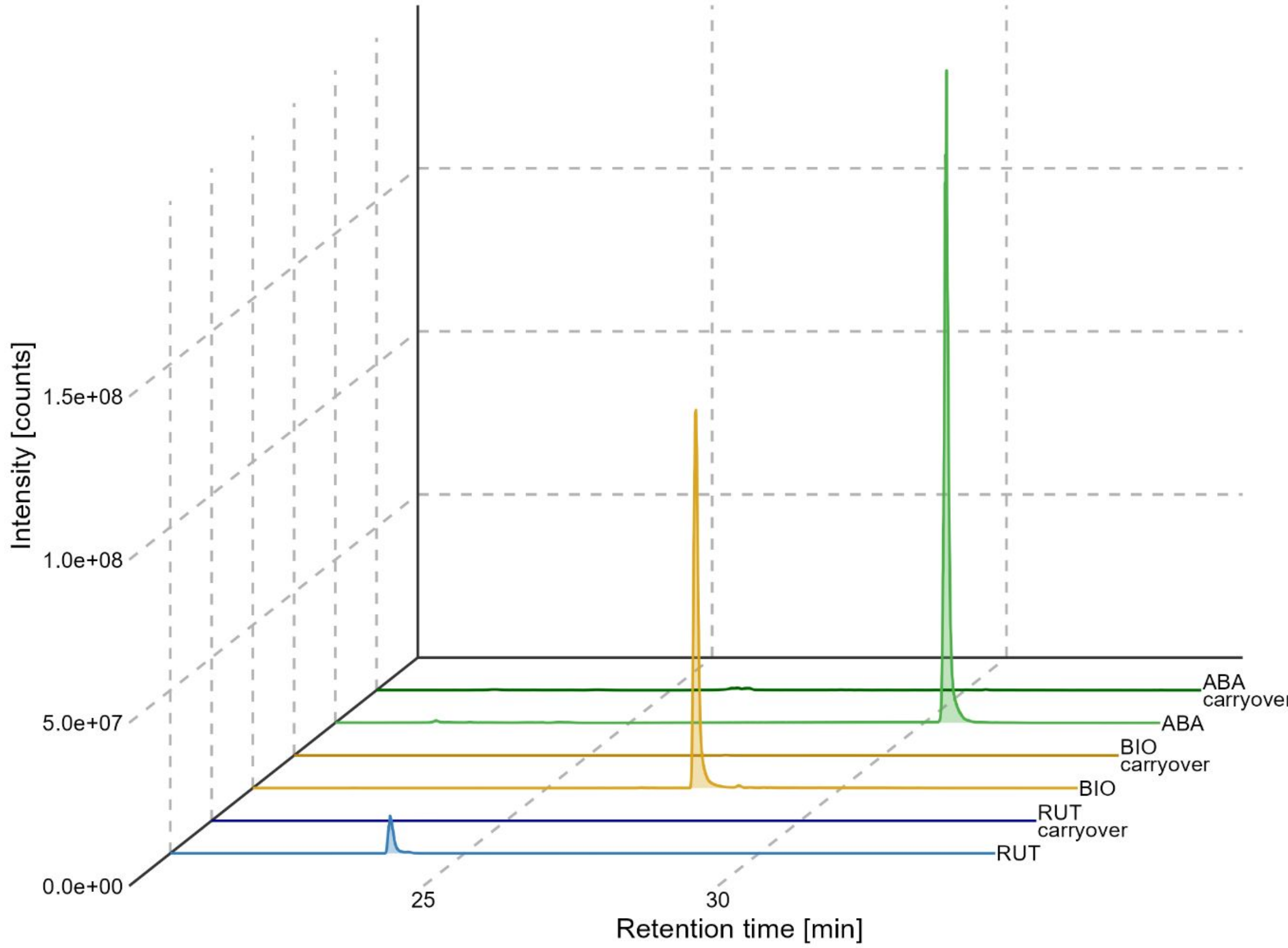

Figure S3. Carryover assessment following a 500 µL large-volume injection of a mixture of Rutin (RUT), Biochanin A (BIO), and Abietic acid (ABA) on an online SPE-UHPLC system. Subsequent blank injections revealed negligible carryover across all three analytes.

*Email: gerd.balcke@ipb-halle.de

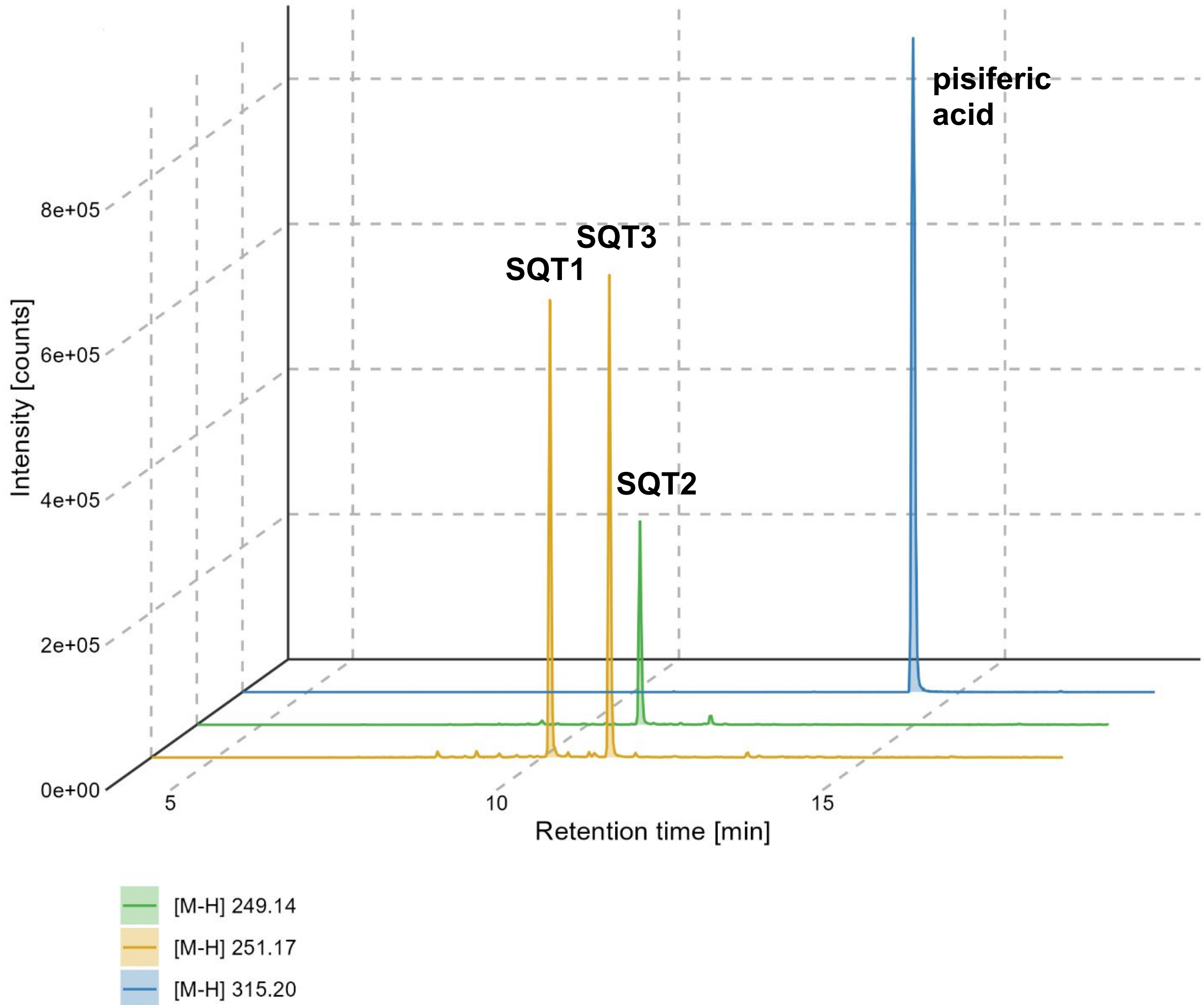

Figure S4. Selected ion chromatograms of three fungal sesquiterpenes (1-3) and Pisiferic acid (spiked reference) from the raw extract (5 µL injection, regular UPLC)

*Email: gerd.balcke@ipb-halle.de

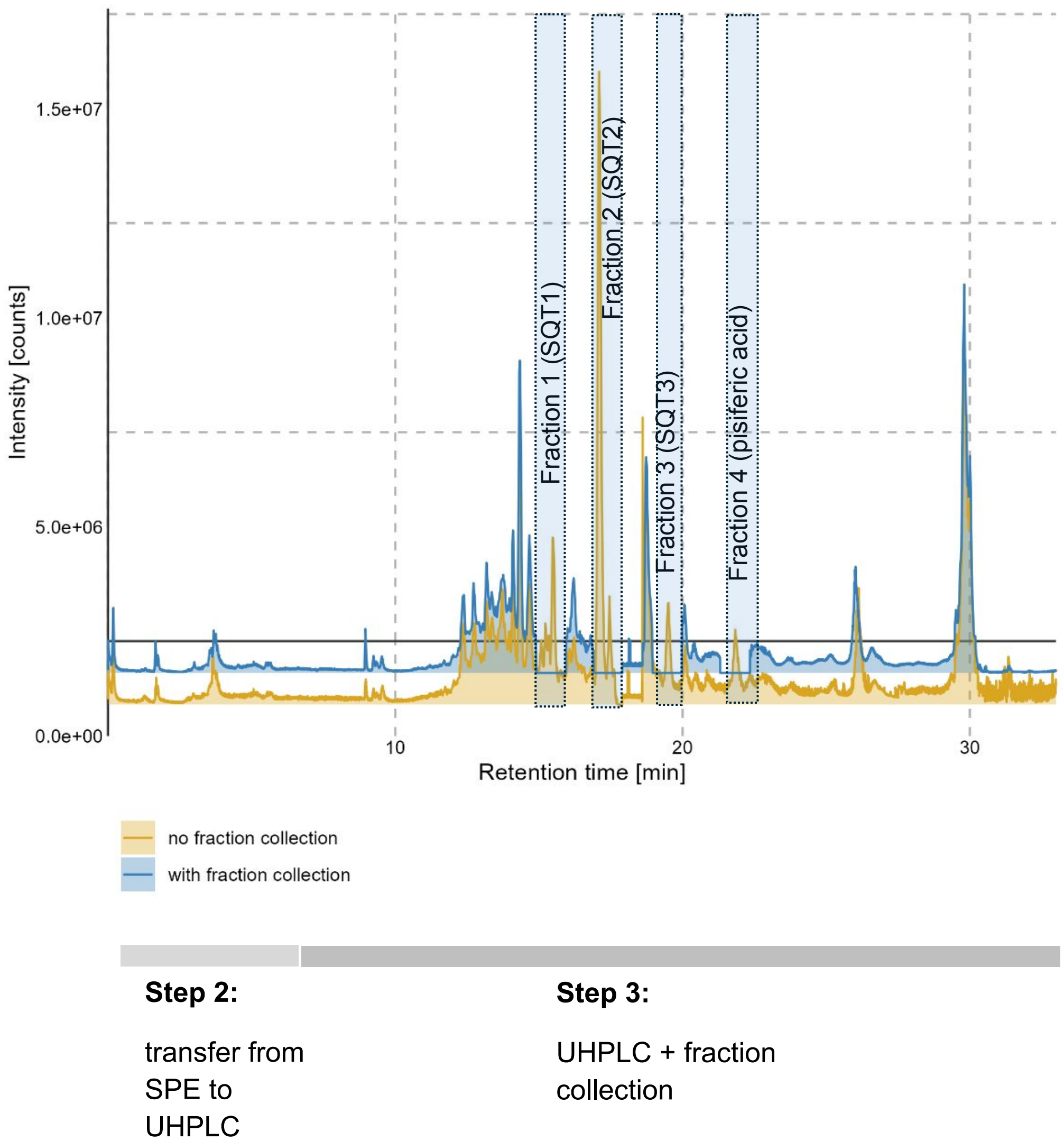

Figure S5. Example for peak purification by micro-SPE-UHPLC-SPE. Fraction collection of sesquiterpenes (1-3) and Pisiferic acid (4). After loading of 400 µL fungal raw extract to online SPE (step 1), the sample was transferred from SPE to UPLC (step 2). Four fractions were collected to receiver SPEs after chromatographic separation by RP-UPLC (step 3). Light red: without fraction collection, light blue: with fraction collection.

*Email: gerd.balcke@ipb-halle.de

Table S2. NMR data of <u>SQT1</u> (CD_3OD, 700 MHz)

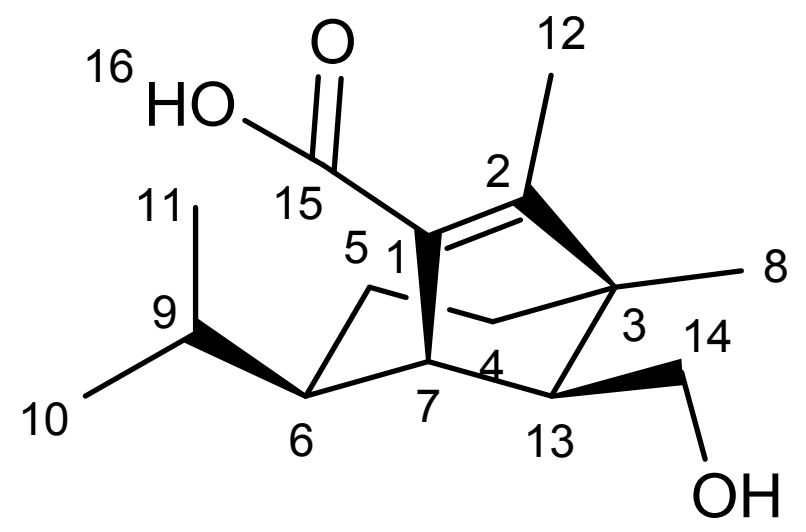

Chemical Formula: $C_{15}H_{24}O_3$

internal reference: HMDSO (^1H = 0.062 ppm)

Pos.	^{13}C: δ [ppm][a]	^1H: δ [ppm] m (J[Hz])	selected HMBC (H → C)	NOESY	COSY
1	128.91	---			
2	157.74	---			
3	50.85	---			
4	35.03	1.34 m	3, 5, 6, 13,	12	
5	26.47	1.01 [b]<br>1.73 m		 4, 9	
6	46.78	1.01 [b]		13	9, 5
7	44.68	3.20 bs		11, 13	6
8	19.21	0.96 s	3, 4, 13	12, 14	
9	33.1	1.21 m			6, 10, 11
10	21.32	0.80 d (6.6)	6, 9, 11		
11	22.38	1.04 d (6.4)		7	
12	12.35	1.95 s	1, 2, 3, 4 6, 7, 15		
13	63.04	1.57 dd (9.6, 5.0)	1, 3, 4, 6, 14	14	
14	62.28	3.28 dd (10.8, 9.6)<br>3.59 dd (10.8, 5.0)			13
15	171.33	---			

[a] ^{13}C chemical shifts based on HSQC correlation peak and quaternary carbon based on HMBC signals

[b] overlapped ^1H-NMR signal, therefore the chemical shift is based on HSQC

*Email: gerd.balcke@ipb-halle.de

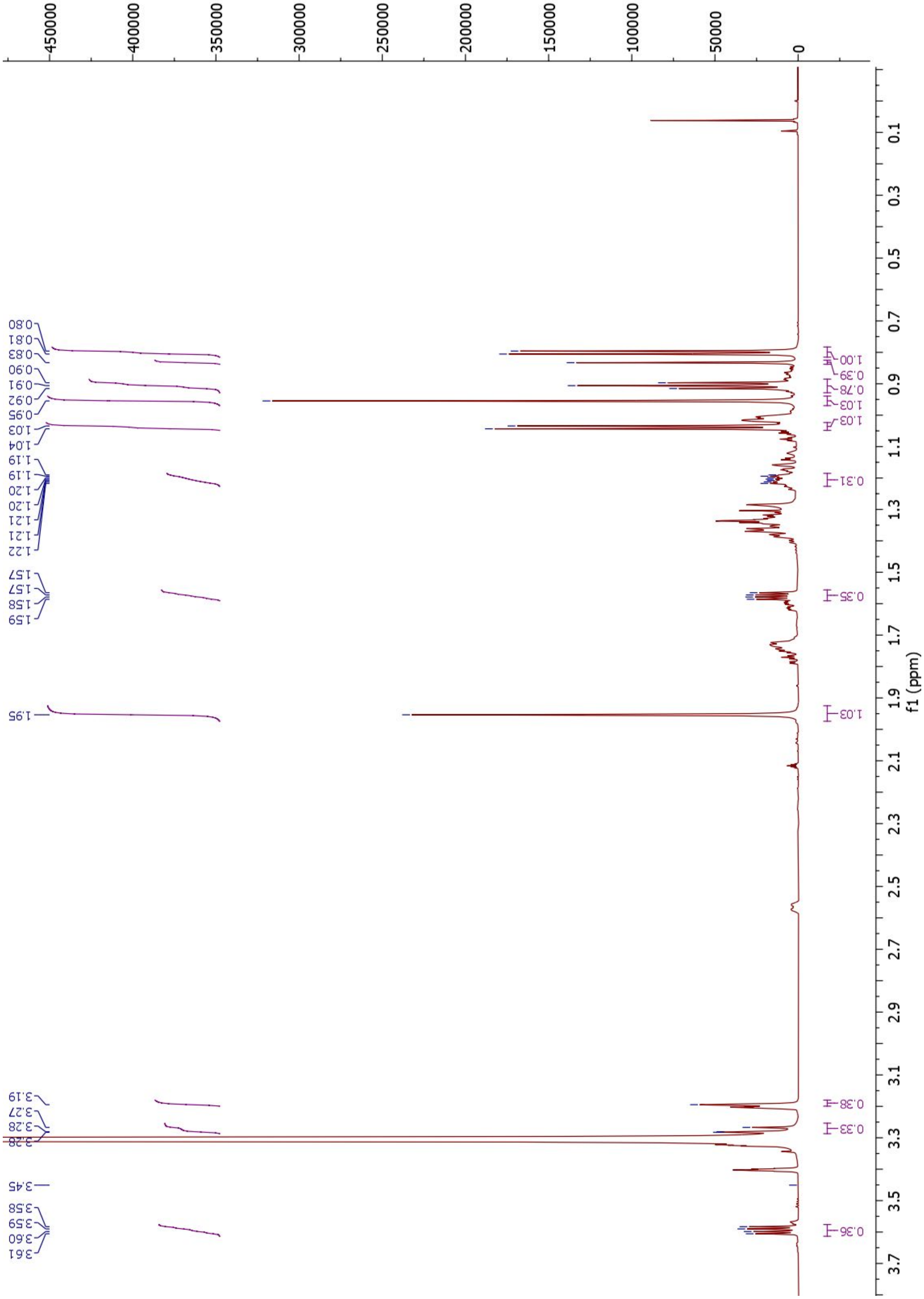

Figure S6. ^{1}H-NMR spectrum of SQT1 (CD_3OD with HMDSO)

*Email: gerd.balcke@ipb-halle.de

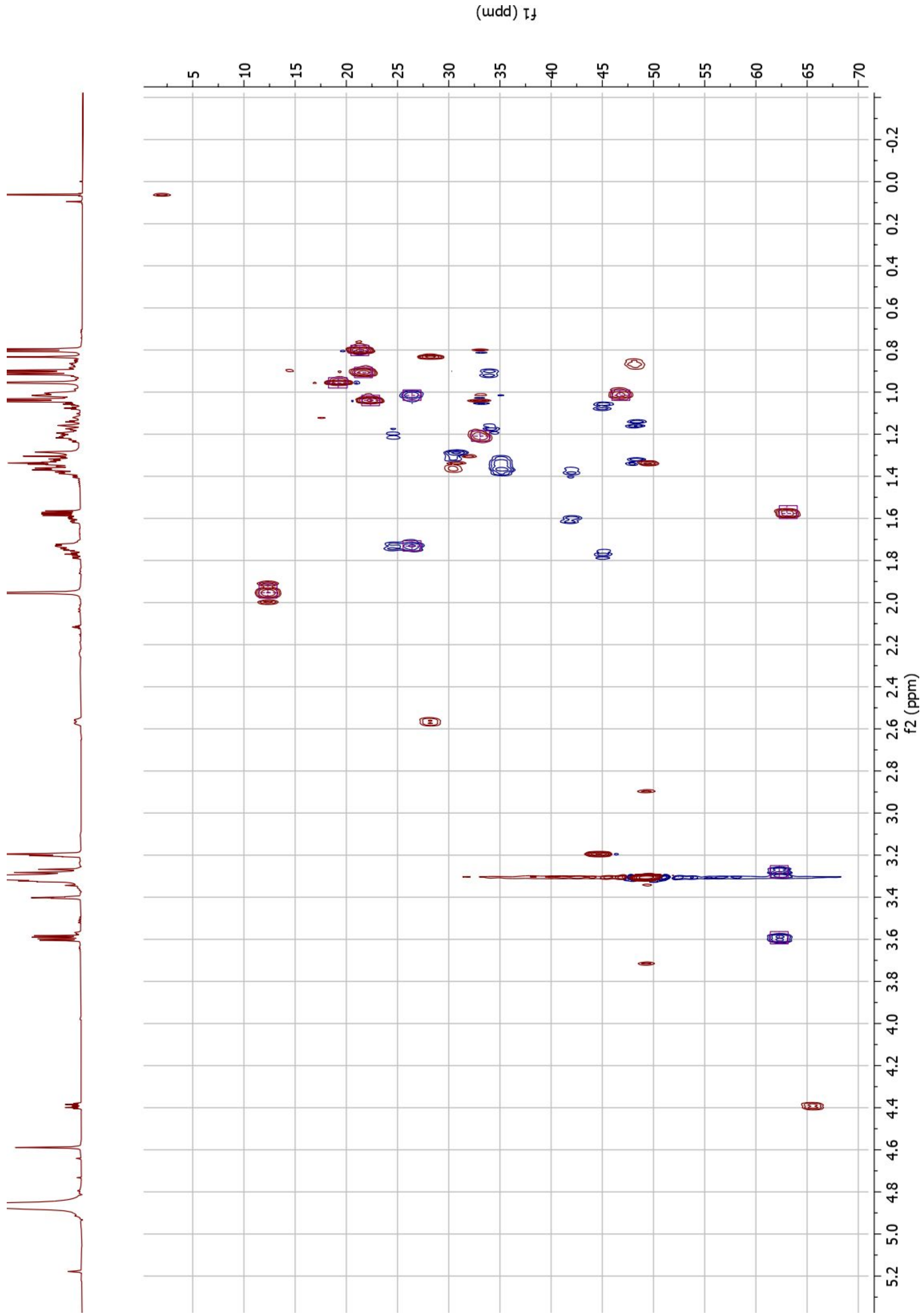

Figure S7. HSQC spectrum of SQT1 (CD_3OD with HMDSO)

*Email: gerd.balcke@ipb-halle.de

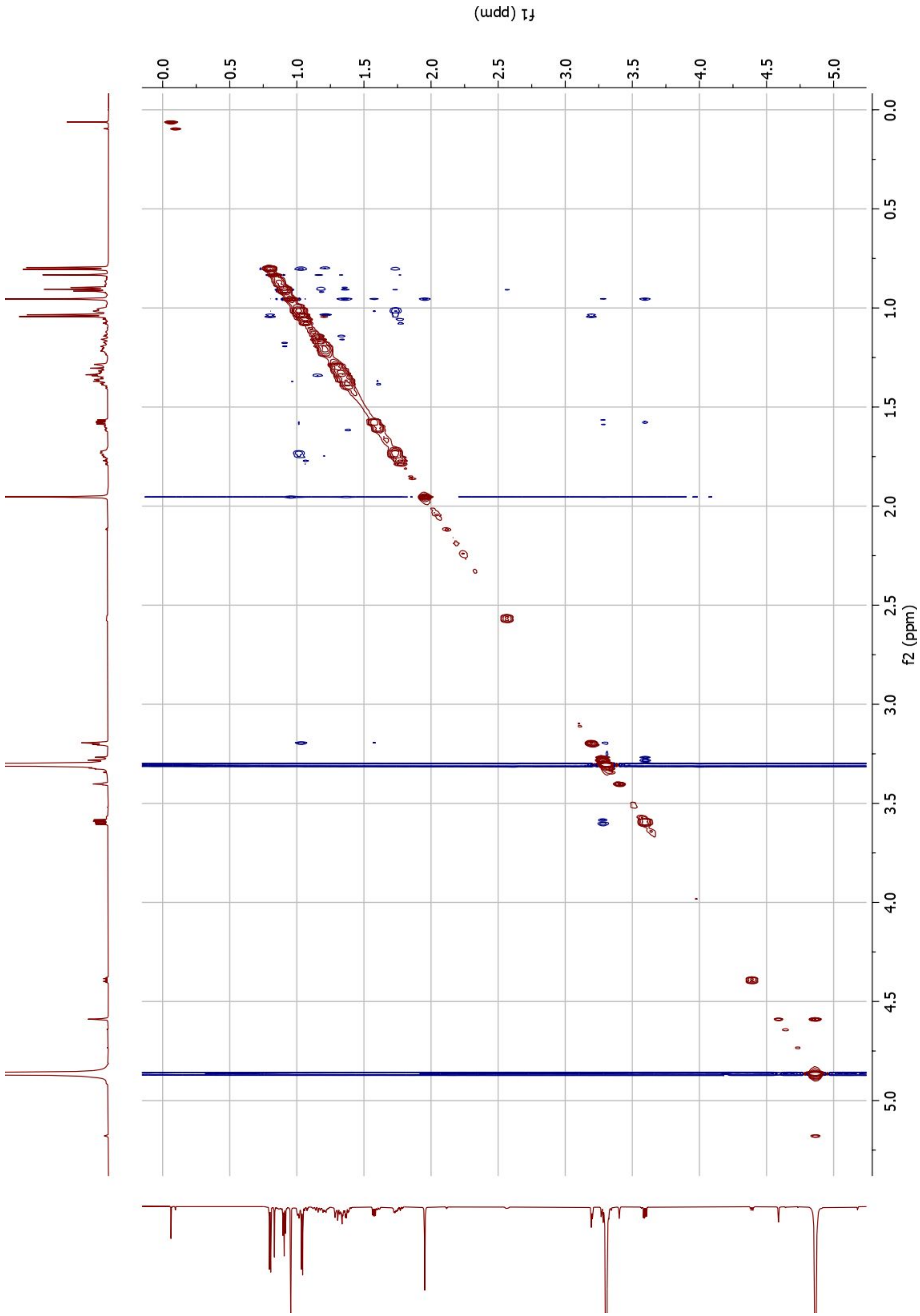

Figure S8. NOESY spectrum of SQT1 (CD_3OD with HMDSO)

*Email: gerd.balcke@ipb-halle.de

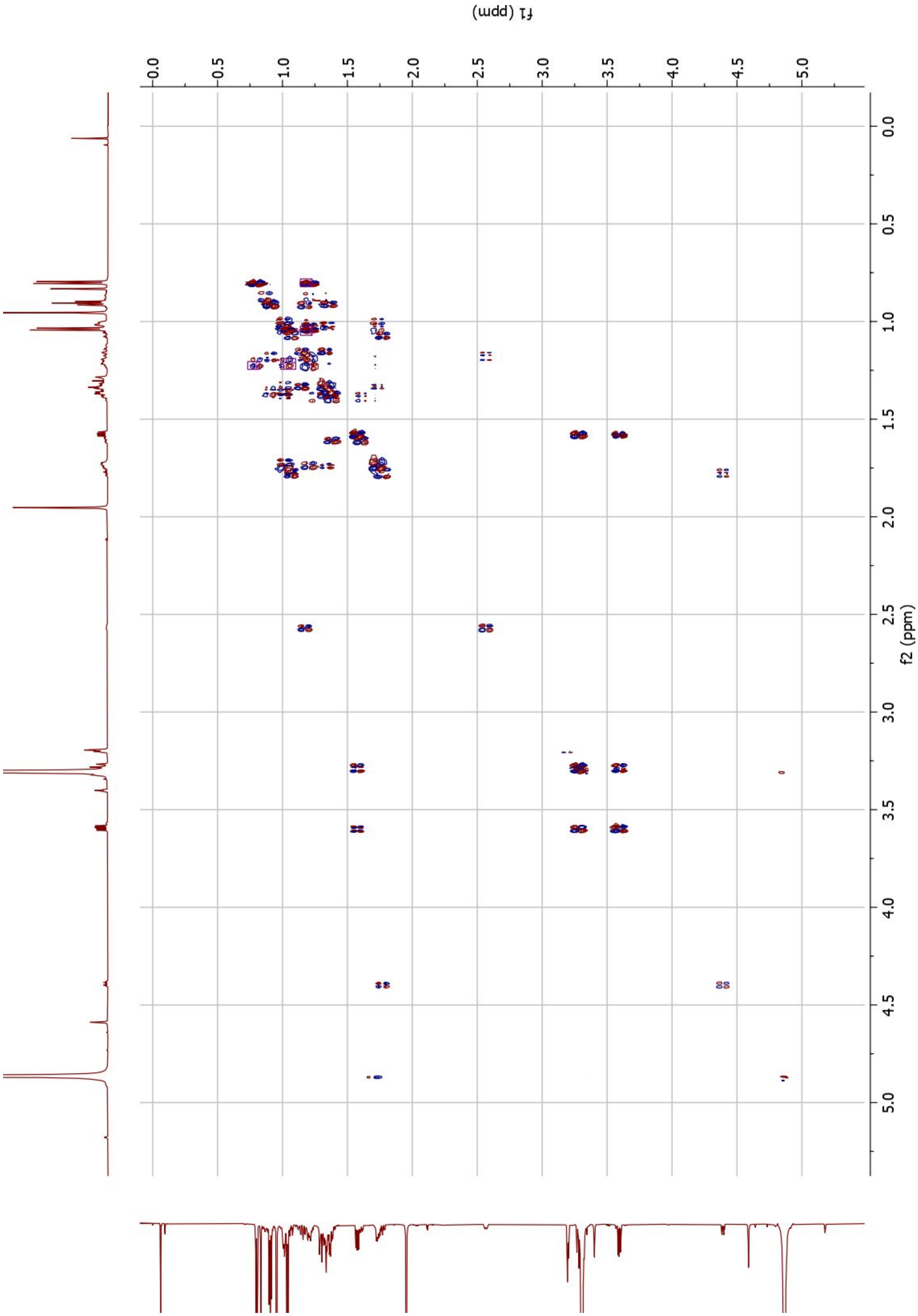

Figure S9. DQCOSY spectrum of SQT1 (CD_3OD with HMDSO)

*Email: gerd.balcke@ipb-halle.de

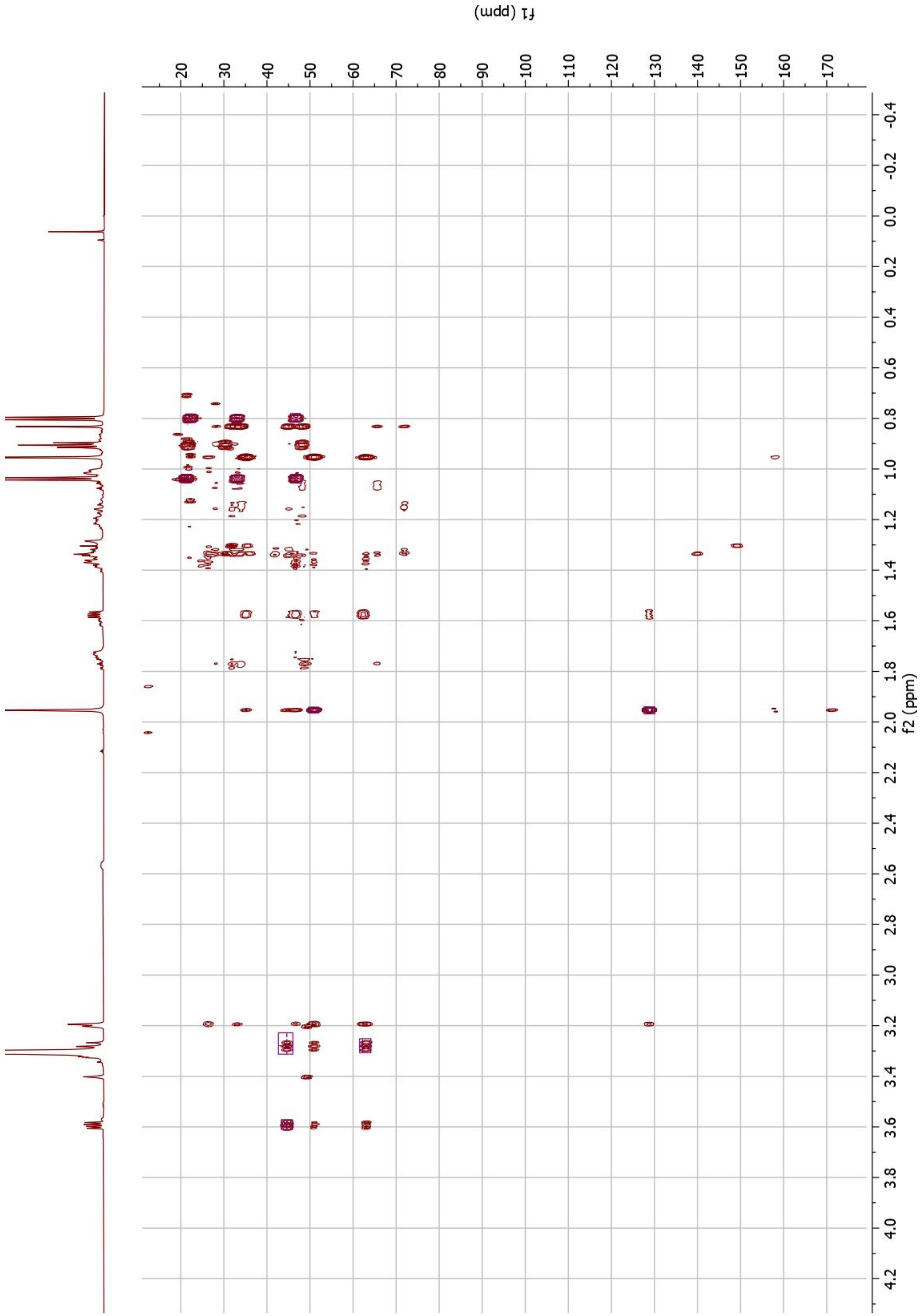

Figure S10. HMBC spectrum of SQT1 (CD$_3$OD with HMDSO)

*Email: gerd.balcke@ipb-halle.de

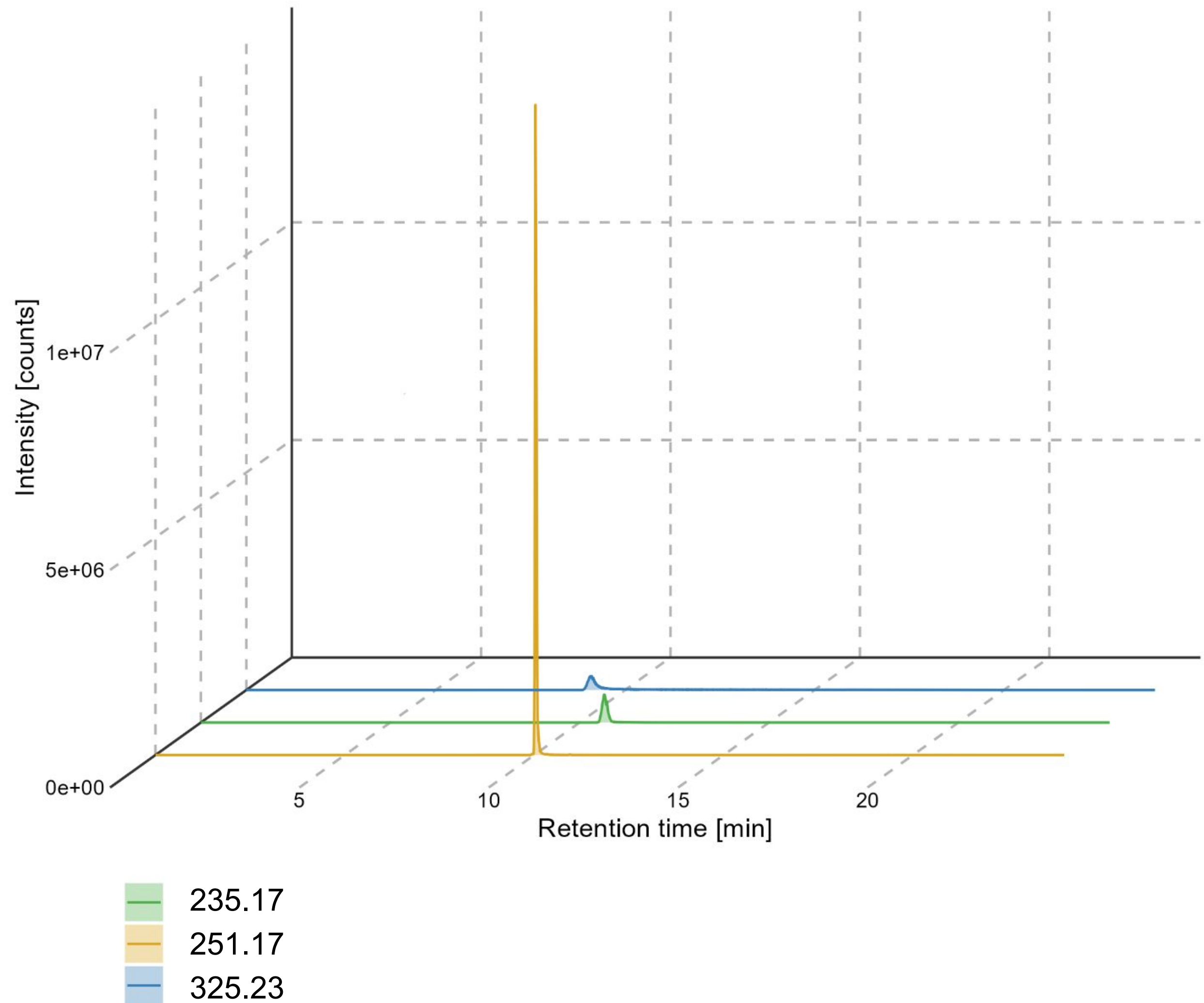

Figure S11. Purity analysis of SQT1 (Helminthosporic acid) after 2D-purification, Yellow [M-H] 251.17 (Helminthosporic acid), green [M+H] 235.17 and blue [M+H] 325.23, which are the only notable contamination detected by regular UPLC (5 µL injection, ESI neg and ESI pos).

*Email: gerd.balcke@ipb-halle.de

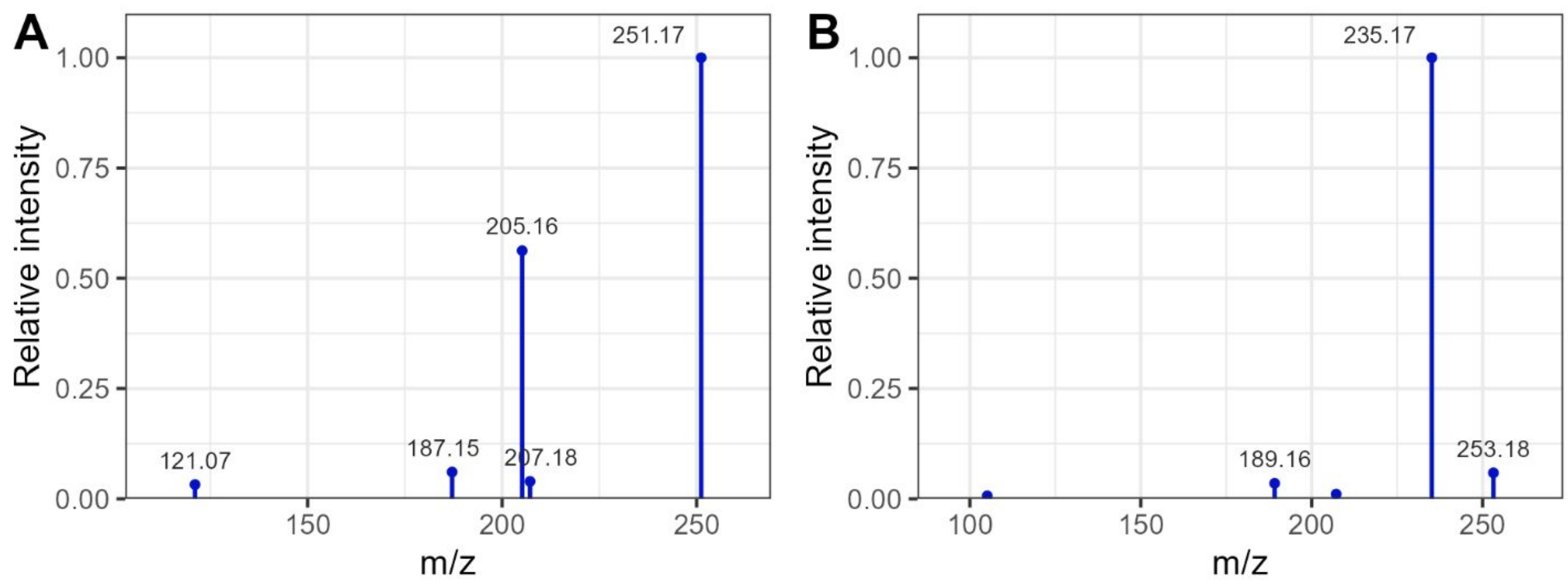

Figure S12. MS/MS spectra of SQT1 (Helminthosporic acid), A – negative ESI, B – positive ESI

*Email: gerd.balcke@ipb-halle.de

Table S3. NMR data of <u>SQT2</u> (C_{D3OD}, 700 MHz)

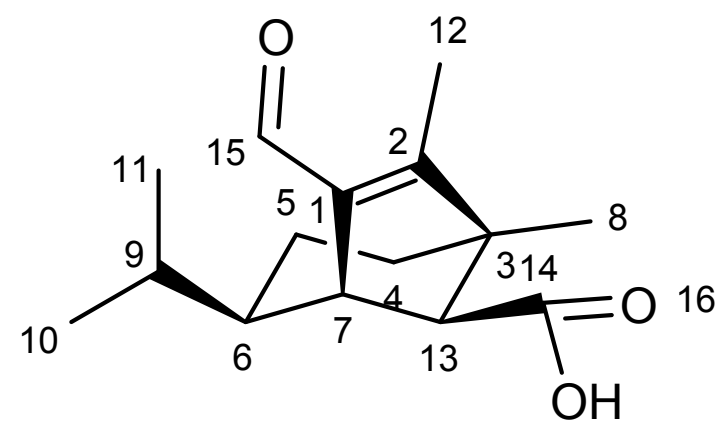

Chemical Formula: $C_{15}H_{22}O_3$

internal reference: HMDSO (^{1}H = 0.062 ppm)

Pos.	^{13}C: δ [ppm][a]	^{1}H: δ [ppm] m (J[Hz])	selected HMBC (H → C)	NOESY
1	138.76	---		
2	167.97	---		
3	52.80	---		
4	34.88	1.51 ddd 13.6, 6.8, 1.4<br>1.45 ddd 13.6, 12.3, 5.9	2,8,5,6,3,13,14	
5	26.24	1.78 m<br>0.92 m	3,4,6	
6	46.20	1.11 m	5, 7,9,10	
7	44.37	3.32[b]	1,2,3,5,6,13,14	
8	19.62	1.19 s	2,3,4,5,13	4, 12,13
9	32.70	1.03 ddd	6,10	
10	21.13	0.79 d (6.3)	11, 9, 6	5 (1.78), 11
11	21.96	1.06 d 5.93	6,9,10	
12	10.67	2.07 s	1,2,3,	15, 5 (0.92); 4 (1.51)
13	65.47	2.36 s	1,2,3,4,6,7, 14	6, 4 (1.45); 8
14	177.50	---		
15	189.8	9.97 s	1, 7	

[a] ^{13}C chemical shifts based on HSQC correlation peak and quaternary carbons based on HMBC signals

[b] overlapped ^{1}H-NMR signal, therefore the chemical shift is based on HSQC

*Email: gerd.balcke@ipb-halle.de

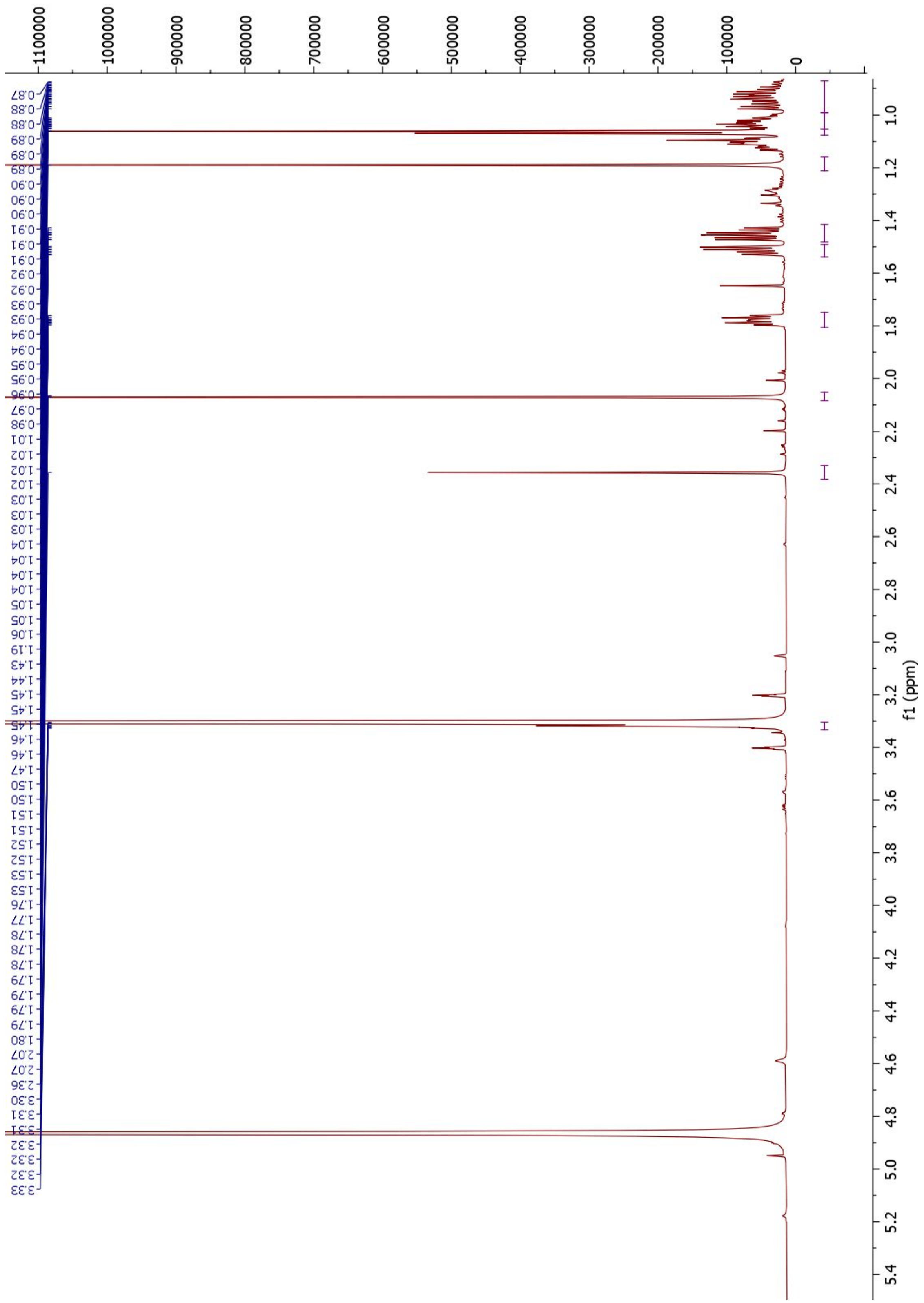

Figure S13. ¹H-NMR spectrum of SQT2 (CD_3OD with HMDSO)

*Email: gerd.balcke@ipb-halle.de

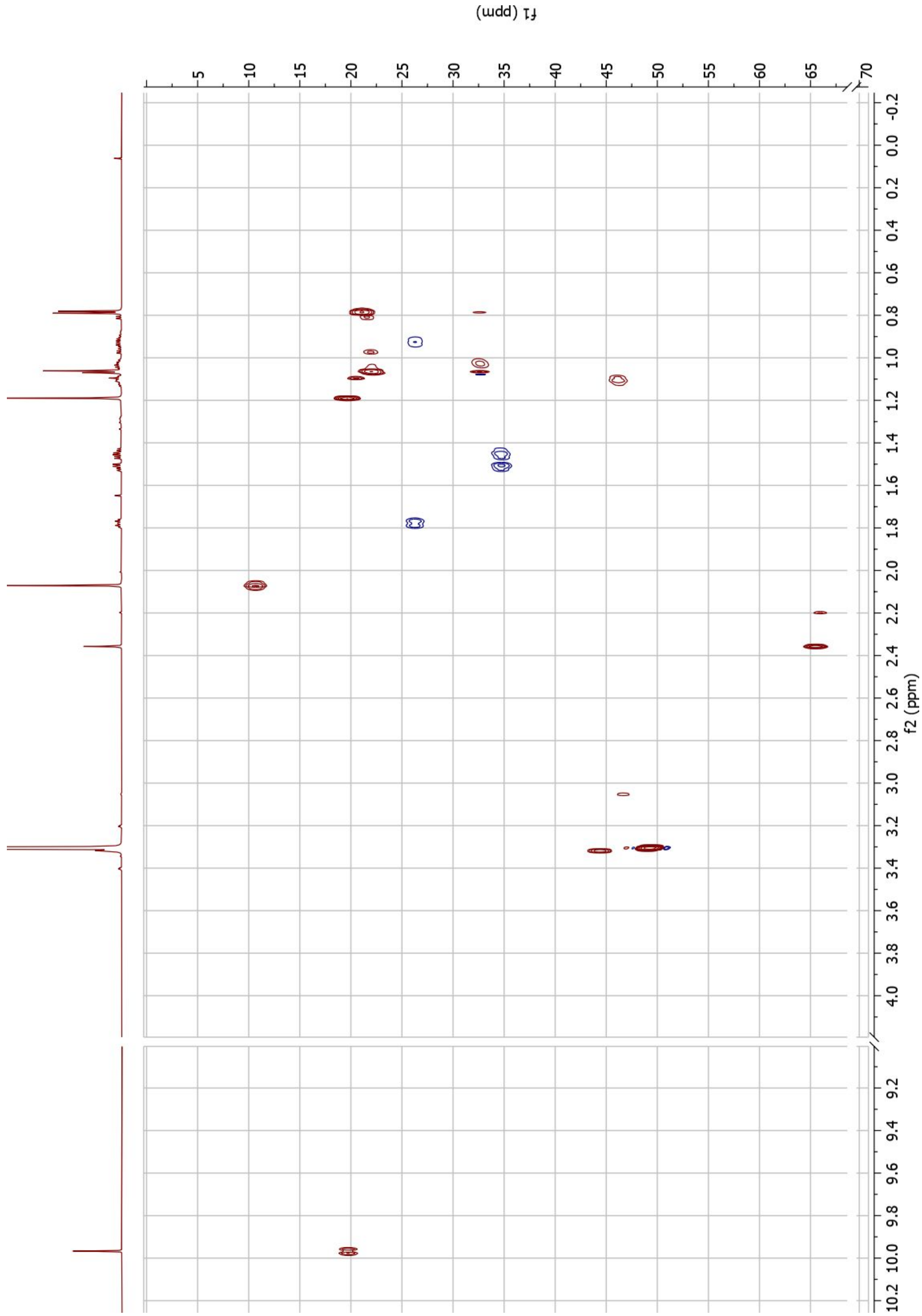

Figure S14. HSQC spectrum of SQT2 (CD_3OD with HMDSO)

*Email: gerd.balcke@ipb-halle.de

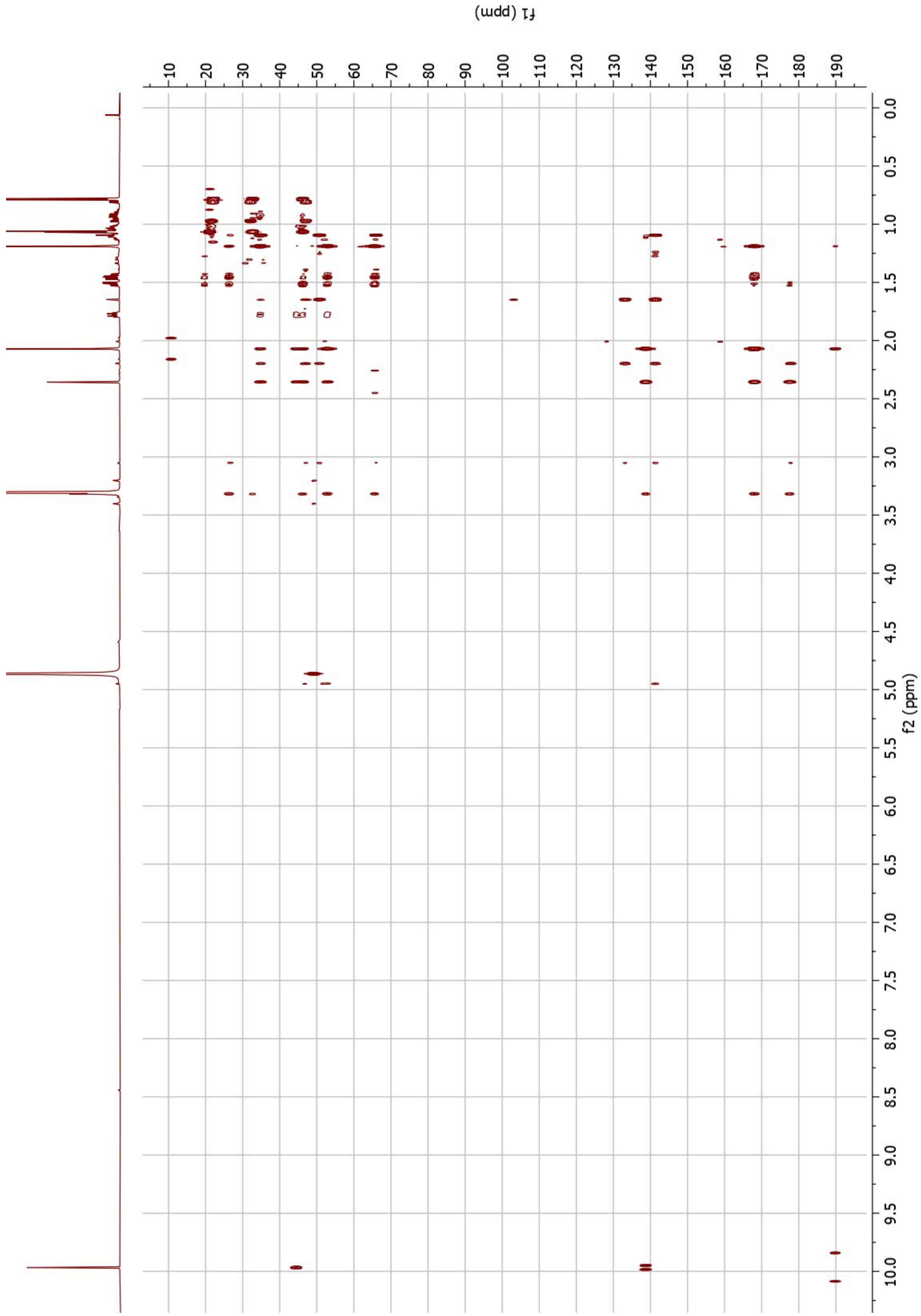

Figure S15. HMBC spectrum of SQT2 (CD_3OD with HMDSO)

*Email: gerd.balcke@ipb-halle.de

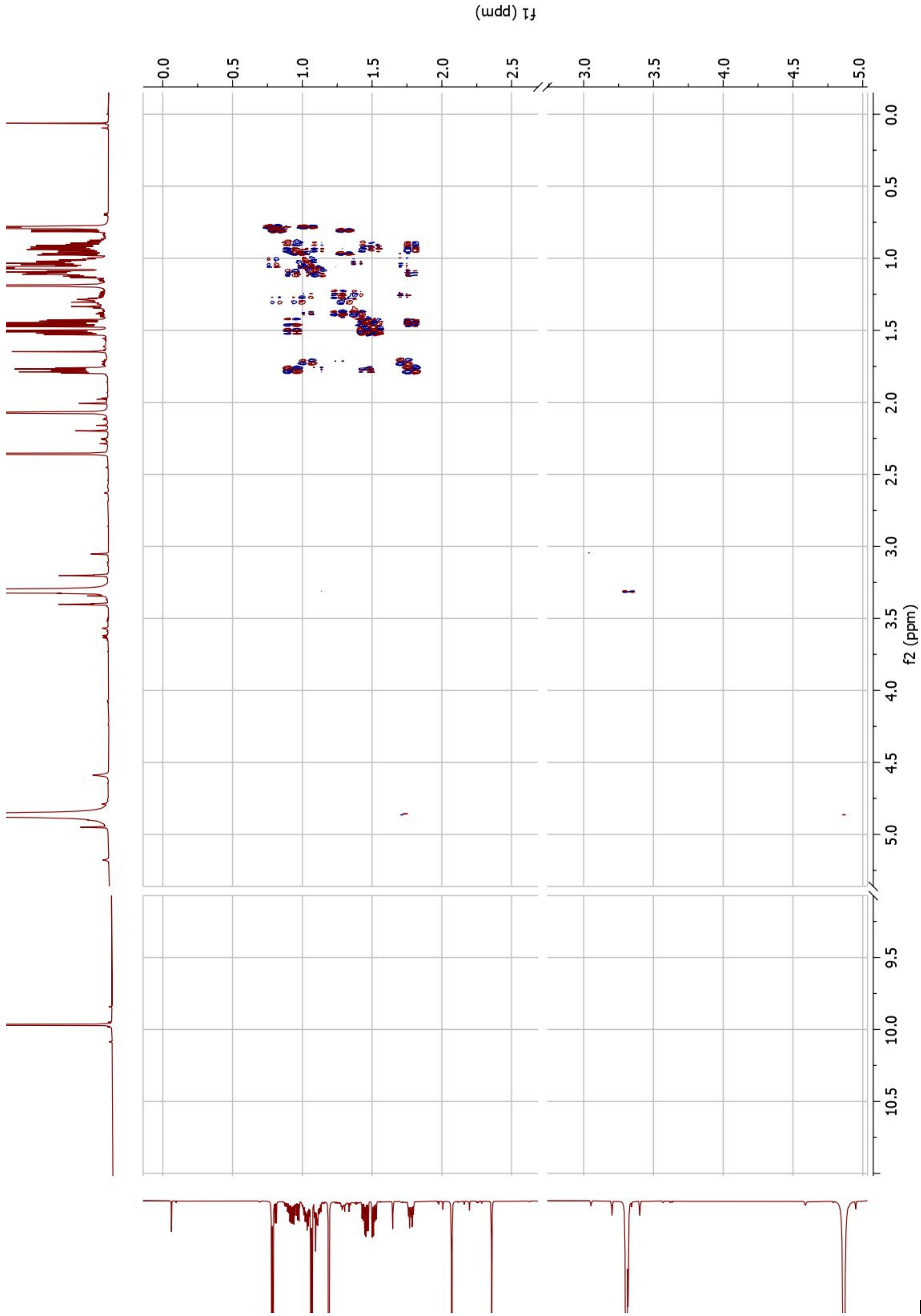

Figure S16. DQCOSY spectrum of SQT2 (CD_3OD with HMDSO)

*Email: gerd.balcke@ipb-halle.de

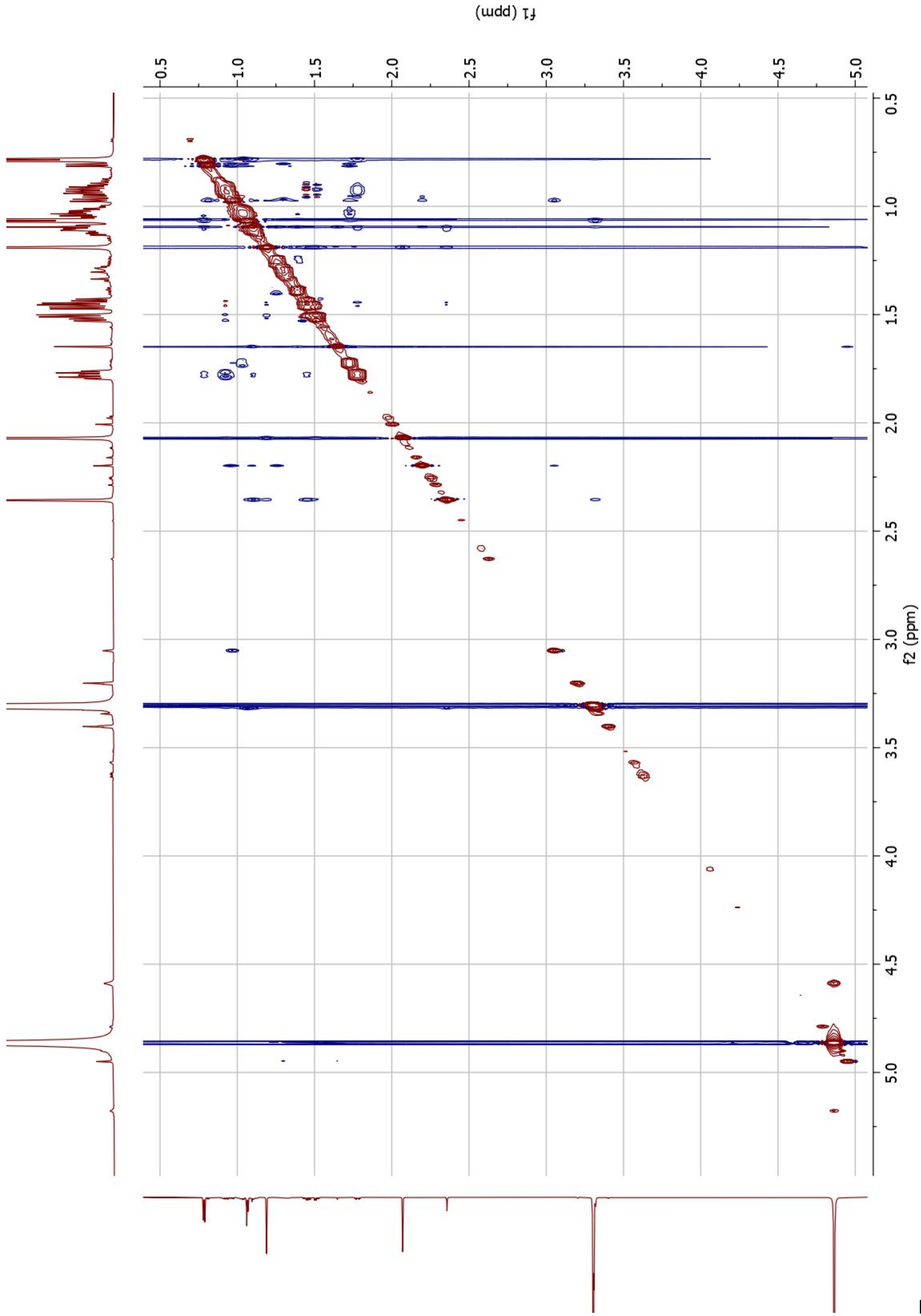

Figure S17. NOESY spectrum of SQT2 (CD_3OD with HMDSO)

*Email: gerd.balcke@ipb-halle.de

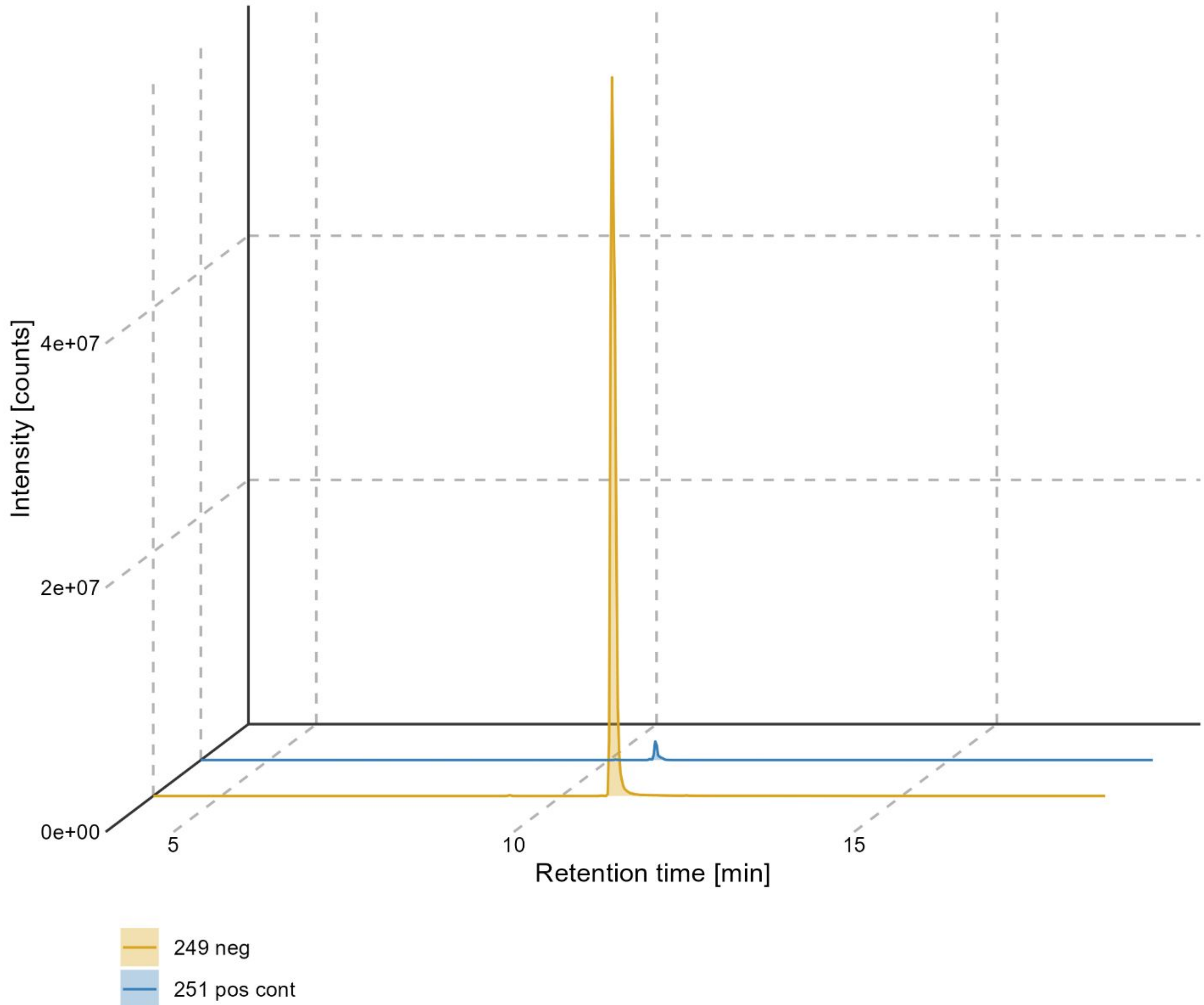

Figure S18. Purity analysis of SQT2 (Helminthosporal acid, [M-H] 249.15) after 2D-purification, [M+H] 251.17 (light blue) is a small contamination only observable in ESI positive ionization mode.

*Email: gerd.balcke@ipb-halle.de

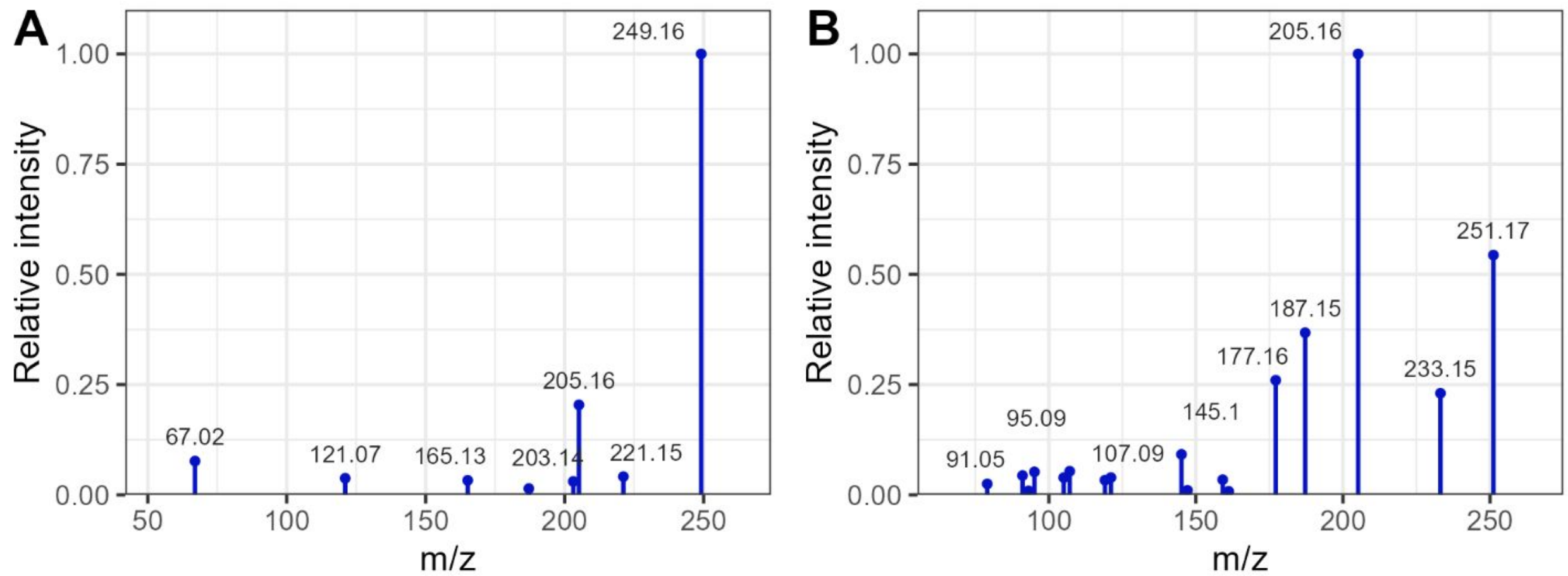

Figure S19. MS/MS spectra of SQT1 (Helminthosporal acid), A – negative ESI, B – positive ESI

*Email: gerd.balcke@ipb-halle.de

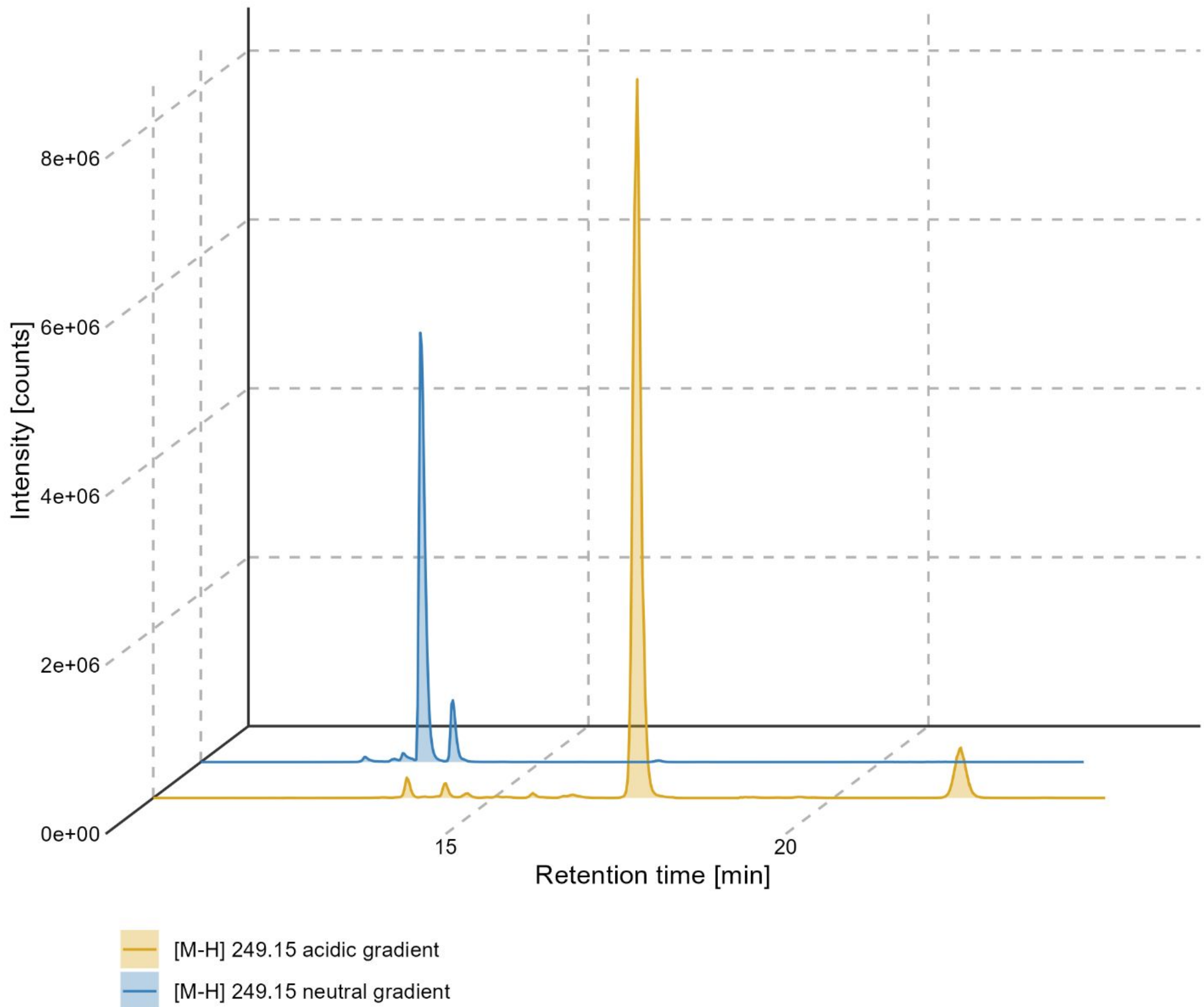

Figure S20: Influence of pH on the retention of Helminthosporal acid (SQT2), yellow: acidic (pH 3.2), blue: neutral (pH 6.7).

*Email: gerd.balcke@ipb-halle.de

Table S4. NMR data of the lactonized SQT3 (CD_3OD, 700 MHz)

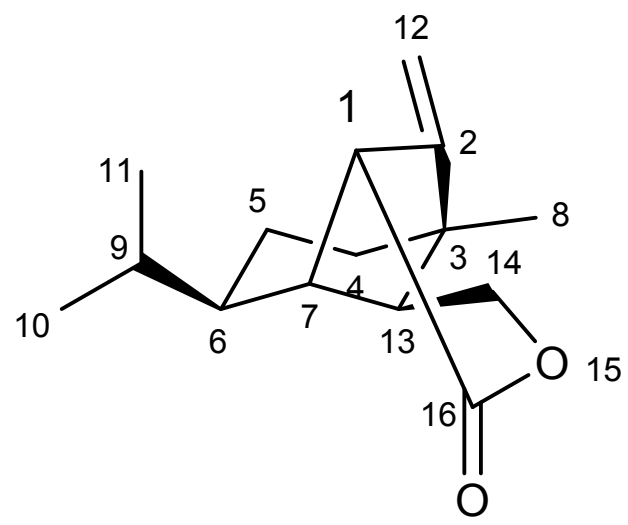

Chemical Formula: $C_{15}H_{22}O_2$
Molecular Weight: 234.34

internal reference: HMDSO (^1H = 0.062 ppm)

Pos.	^{13}C: δ [ppm][a]	^1H: δ [ppm] m (J[Hz])	selected HMBC (H → C)	NOESY	COSY
1	52.21	3.33 m[b]			
2	155.36	---			
3	46.04	---			
4	42.41	1.62 m<br>1.47 ddd (12.8, 5.6, 2.0)	2,3,5,13		5
5	26.45	1.16,<br>1.77			4 (1.62), 6
6	47.15	1.37 m[b]		5, 10, 11, 13	5
7	41.08	2.50 bd (1.76)		1, 6, 11, 14	6
8	20.33	1.24 s	2, 3, 4, 13	14	
9	32.12	1.41 m[b]			10, 11
10	21.16	0.91 d (6.40)	6, 9, 11	5, 1	
11	21.32	0.96 d (6.40)	6, 9, 10	7	
12	106.17	5.14 bs<br>4.89 bs	1, 2, 3, 16	1	
13	50.82	1.88 m		8	14(4.28)
14	71.53	4.29 dd (11.7, 4.6)<br>4.47 dd (11.7, 0.6)	13, 3, 7, 16	7, 13<br>8, 13	
16	174.50	----			

*Email: gerd.balcke@ipb-halle.de

[a] ^{13}C chemical shifts based on HSQC correlation peak and quaternary carbon based on HMBC signals. [b] overlapped ^{1}H-NMR signal, therefore the chemical shift is based on HSQC

*Email: gerd.balcke@ipb-halle.de

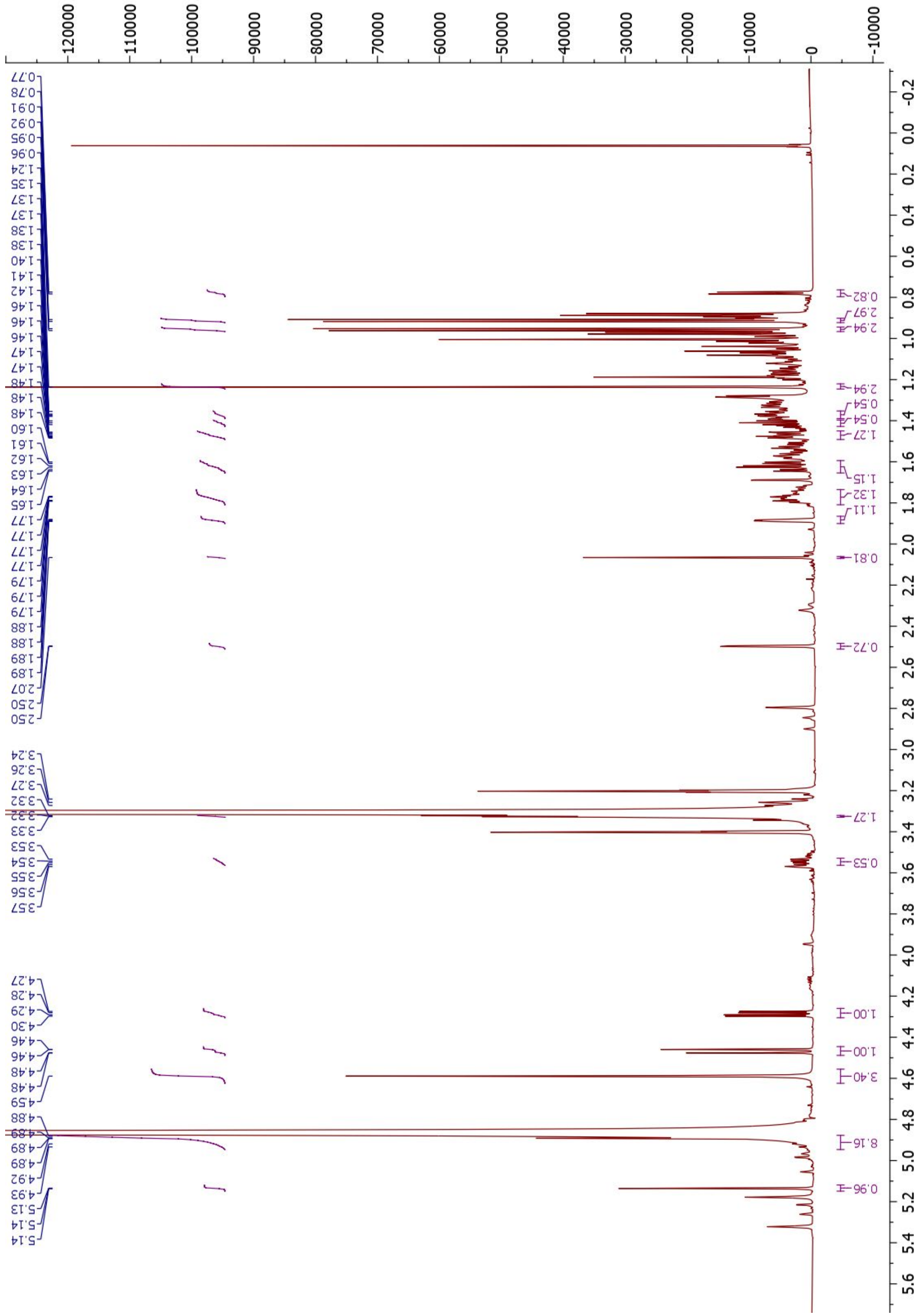

Figure S21. ^{1}H-NMR spectrum of SQT3 (CD_3OD with HMDSO)

*Email: gerd.balcke@ipb-halle.de

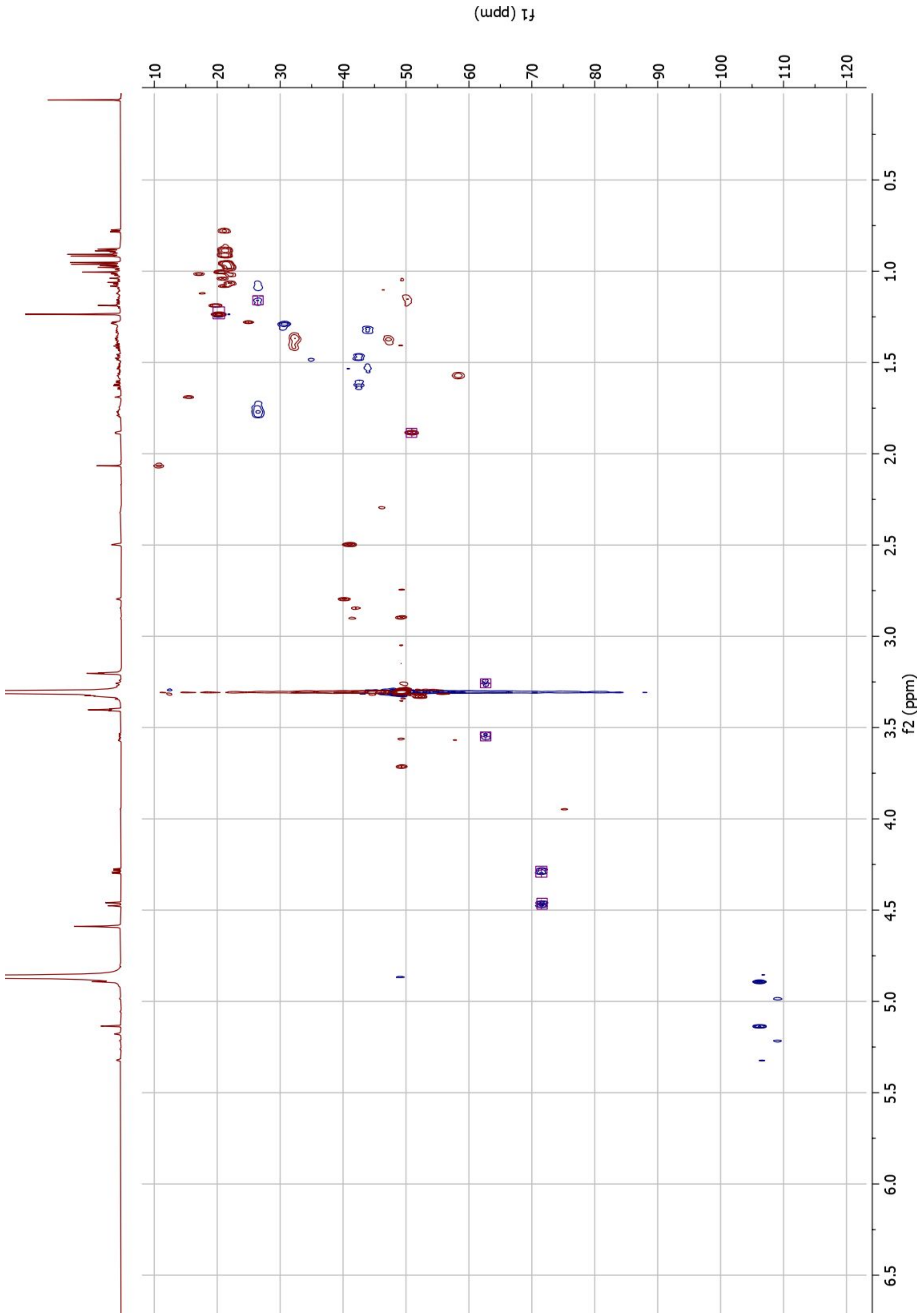

Figure S22. HSQC spectrum of SQT3 (CD_3OD with HMDSO)

*Email: gerd.balcke@ipb-halle.de

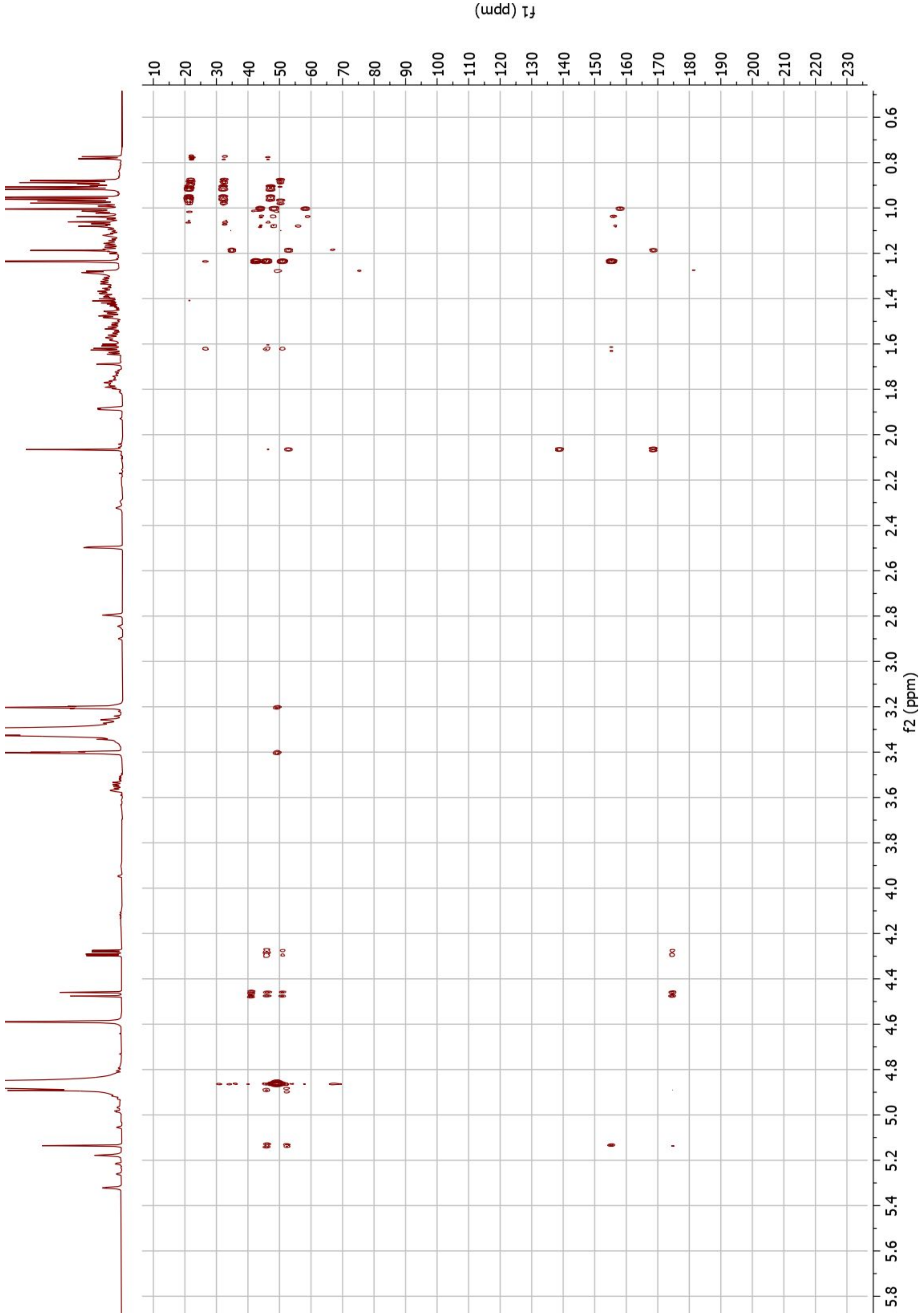

Figure S23. HMBC spectrum of SQT3 (CD_3OD with HMDSO)

*Email: gerd.balcke@ipb-halle.de

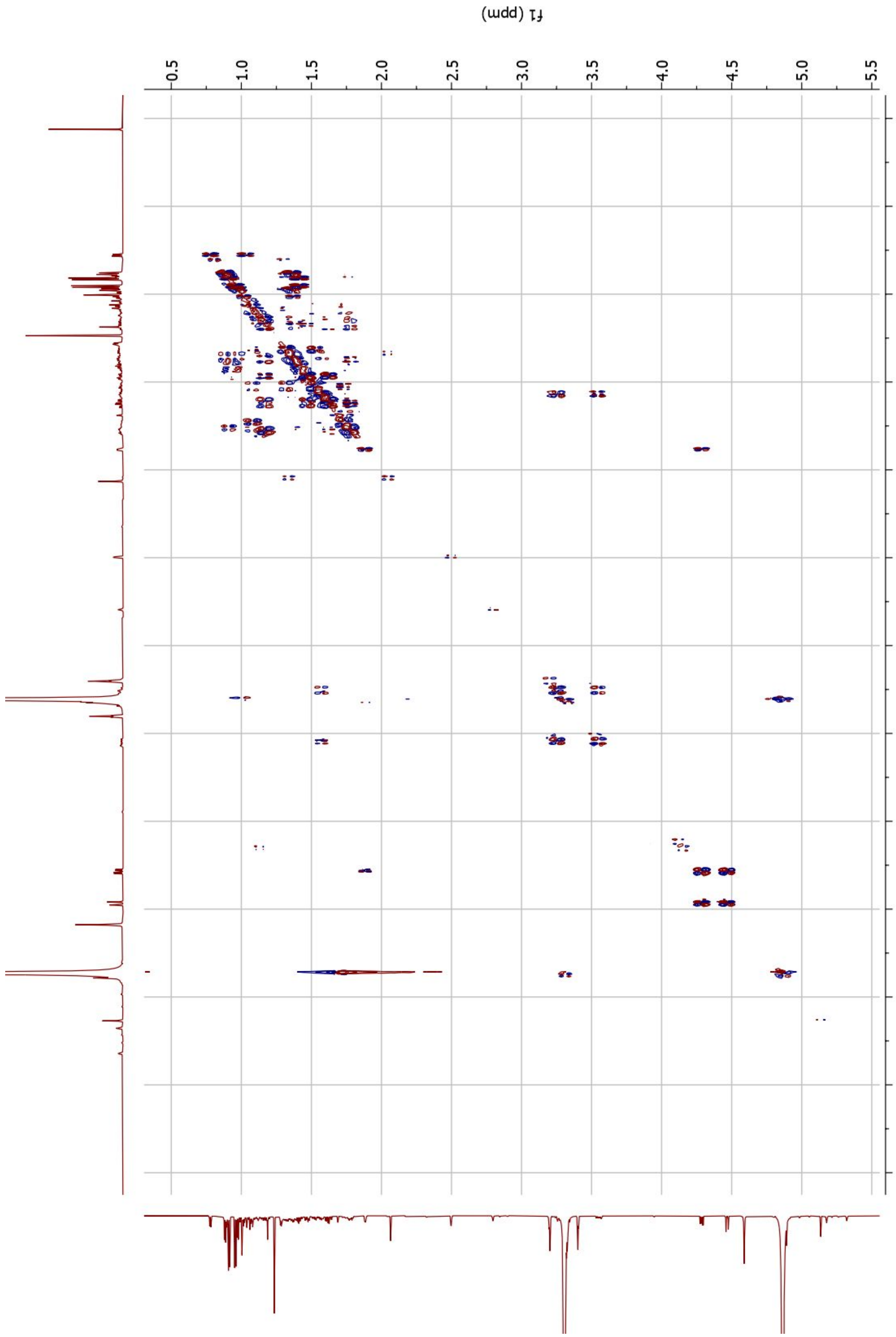

Figure S24. DQCOSY spectrum of SQT3 (CD_3OD with HMDSO)

*Email: gerd.balcke@ipb-halle.de

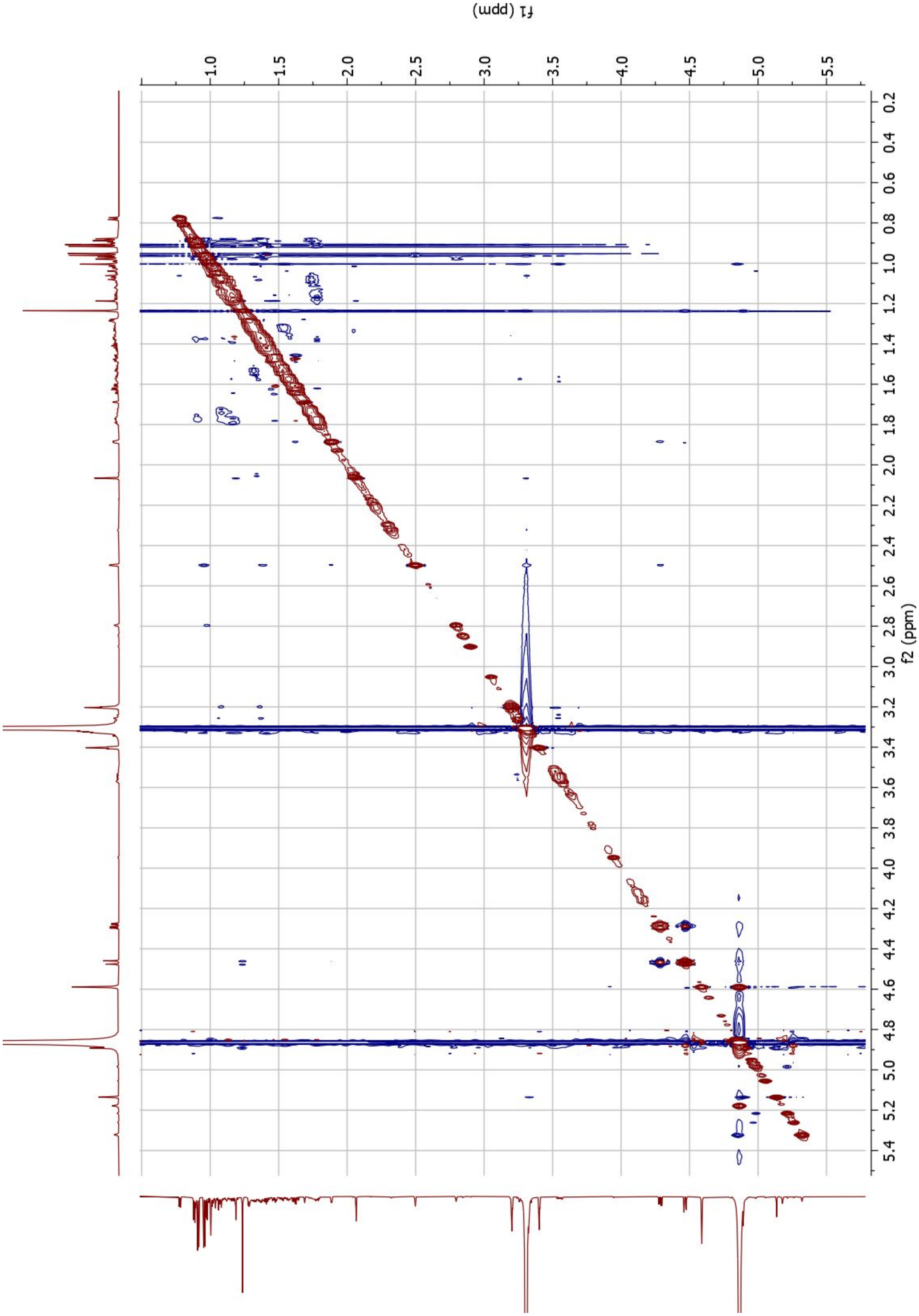

Figure S25. NOESY spectrum of SQT3 (CD_3OD with HMDSO)

*Email: gerd.balcke@ipb-halle.de

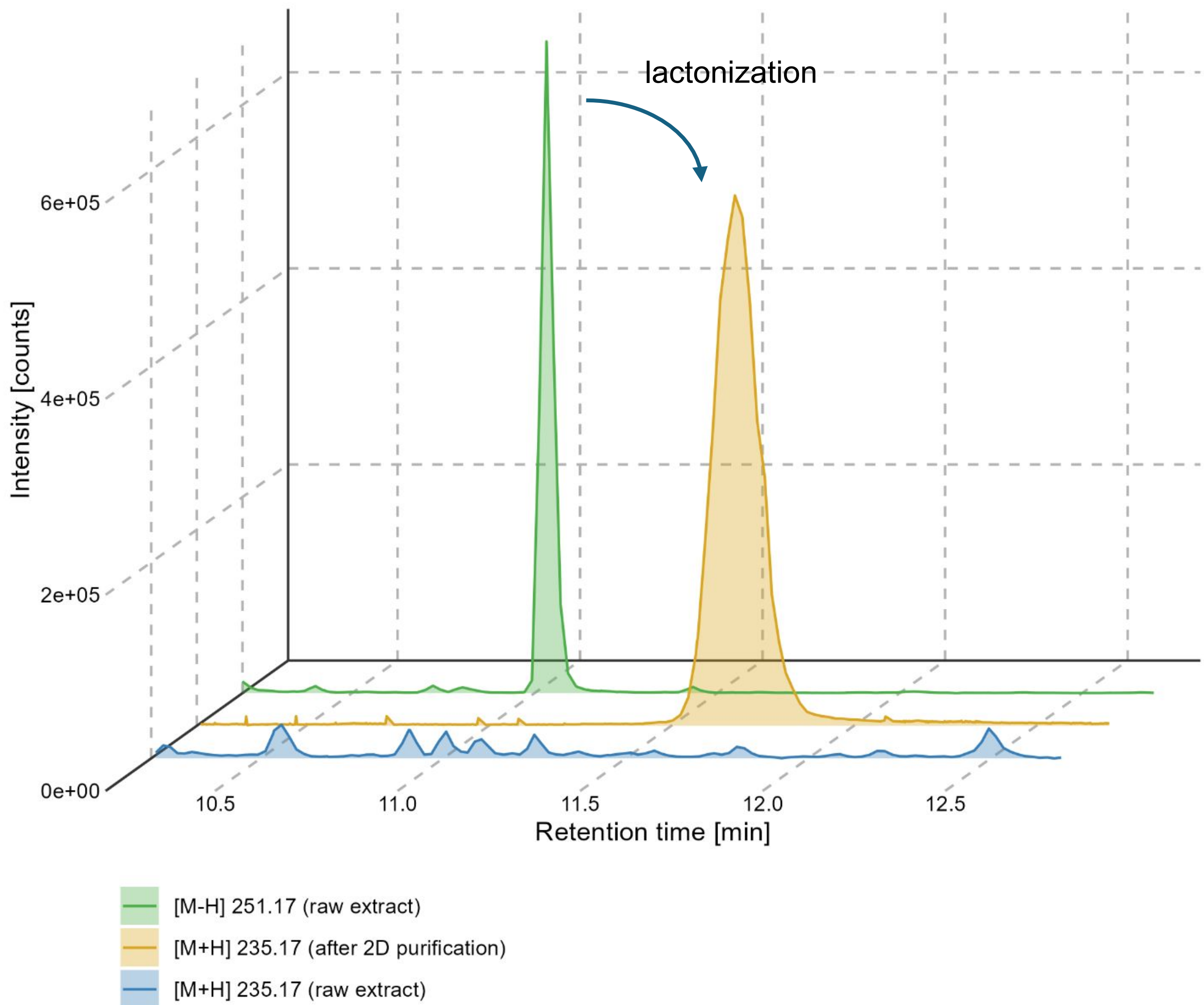

Figure S26. SQT3 [M-H] 251.17, 10.9 min from the raw extract (green) and after purification (blue) illustrates its depletion and the formation of a lactonized product of [M+H] 235.17; 11.7 min (yellow) after 2D-purification, drying and quality analysis by regular RP-UPLC.

*Email: gerd.balcke@ipb-halle.de

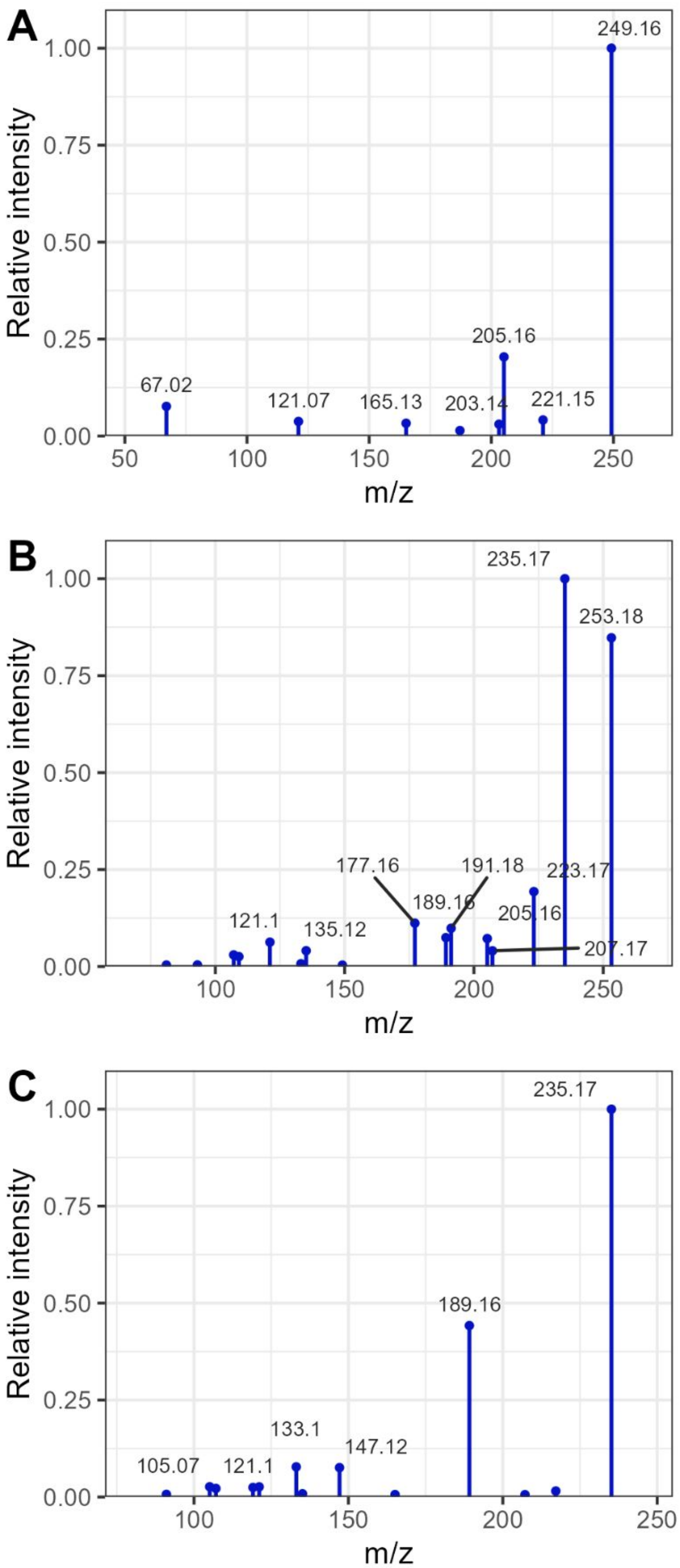

Figure S27. MS/MS spectra of SQT3 (A, negative ESI, B positive ESI) and its lactonized transformation product (panel C, only detectible after positive ESI)

*Email: gerd.balcke@ipb-halle.de

Table S5. Integration results of qNMR study from SQT1, SQT2 and SQT3.

		Integral values				Amount [mg]
	Integral width[a]	1.97 - 1.93	0.97 - 0.94	0.81 - 0.78	0.07 - 0.05	
SQT1	Measurement 1	21023512.54	21892726.87	21618646.64	3173948.77	0.125
	Measurement 2	23887179.88	25188721.05	24691384.57	3565104.95	0.127
	Measurement 3	23655949.32	24677518.99	24280483.87	3550277.21	0.125
	number of protons	3	3	3	18	
					Mean amount and standard deviation:	0.125±0.003
	Integral width[a]	9.98 - 9.96	2.10 - 2.04	1.22 - 1.16	0.07 - 0.05	
SQT2	Measurement 1	37614797.81	115362487.2	118098931.6	3581928.54	0.598
	Measurement 2	36908792.32	113586241.9	116764152.7	3632180.54	0.583
	Measurement 3	36096497.55	111846833.5	115586301.6	3567956.04	0.588
	number of protons	1	3	3	18	
					Mean amount and standard deviation:	0.574±0.015
	Integral width[a]	4.50 - 4.44	4.31 - 4.26	1.25 - 1.22	0.07 - 0.05	
SQT3	Measurement 1	1825311.25	1854485.69	6489529.61	3076596.59	0.036
	Measurement 2	2134578.51	2012106.9	7279288.89	3547218.14	0.035
	Measurement 3	2129737.44	2057921.01	7741006.03	3532498.74	0.037
	number of protons	1	1	3	18	
					Mean amount and standard deviation:	0.032±0.003

[a] Integral width does not include the 13C satellites

*Email: gerd.balcke@ipb-halle.de

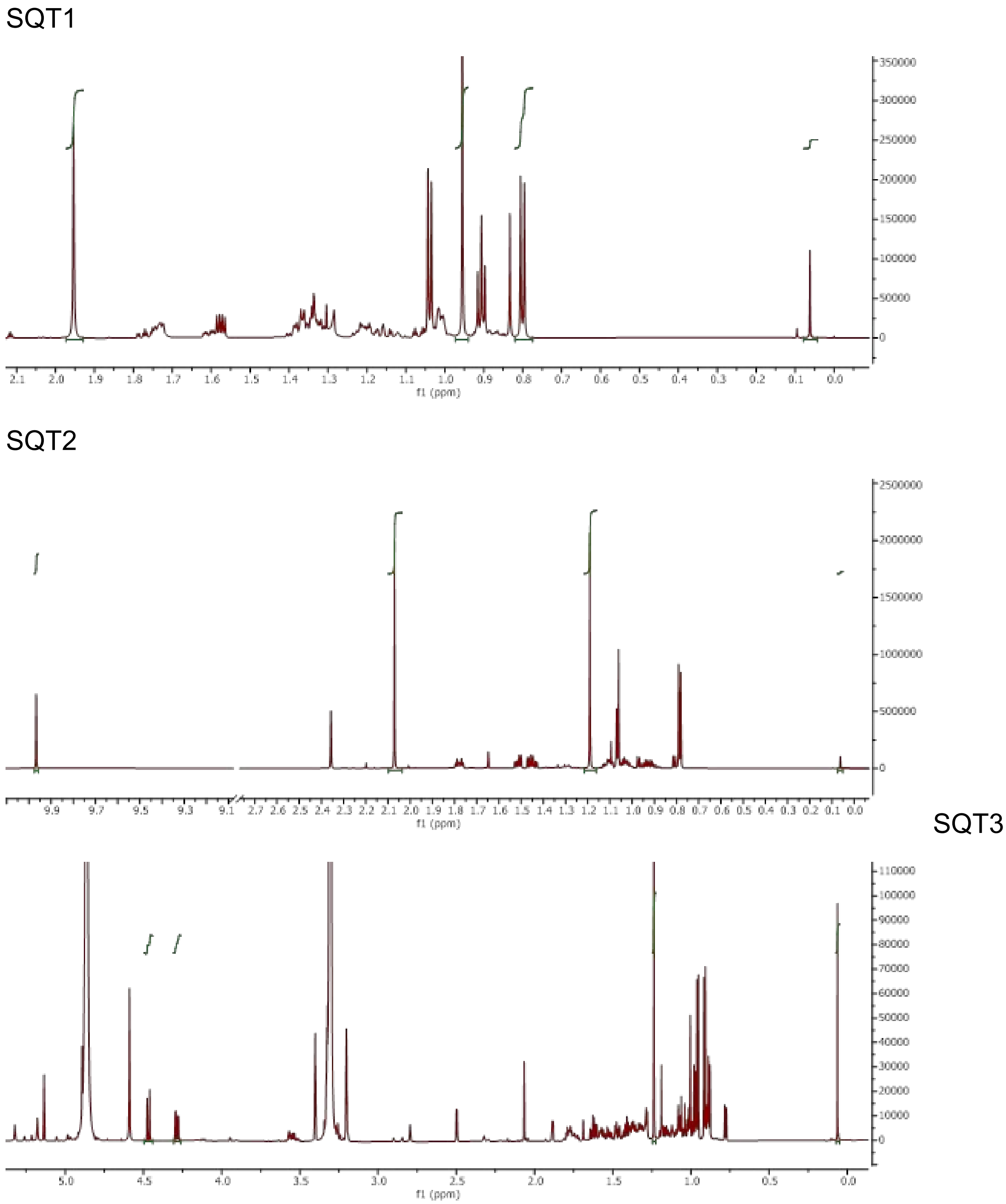

Figure S28. ^{1}H qNMR spectra with chosen signals and integrals for each quantified compound. (CD_3OD with HMDSO, 700MHz)

Table S6. Quantitative comparison of the present online microSPE-UPLC-SPE method with conventional and existing online purification platforms

*Email: gerd.balcke@ipb-halle.de

Metric	Preparative HPLC	Offline SPE + HPLC	UHPLC-SPE-NMR (Bhatia et al.)	This work
Injection volume	0.1 - 5 mL	Manual loading	1–10 µL	Up to 1000 µL (automated)
Solvent consumption per run	100–2000 mL	50–500 mL	~5–20 mL	~30 mL
Time per compound	Days - weeks	Days	Hours - days	<24 h
Recovery (semi-polar compounds)	70–95%	Variable	>80%	86–103%
Automation	Partial	Minimal	Partial	Full
Volatile compound compatibility	Poor (drying required)	Poor	Yes (micro-SPE)	Yes
2D orthogonal purification	Manual	Manual	No	Fully automated
SPE cartridge reuse	N/A	Limited	Limited	Maximum reusability (after conditioning)

N/A, not applicable

Video 1. Video animation illustrating the individual steps of online microSPE-UHPLC-SPE: Step 1 - Large volume sample injection by a high-pressure dispenser (HPD) with simultaneous dilution using a dilution pump; Step 2 - Desorption from SPE cartridge and peak refocussing on UHPLC column head; Step 3 – Peak separation by UHPLC combined with MS-analysis; Step 4 - Post-column fraction collection to microSPE cartridges with simultaneous dilution; Step 5 – Automated fraction desorption to receiving vials.

The last two slides depict the full concept and illustrate the opportunity to increase the peak purity by repeating the process via 2D chromatography.

*Email: gerd.balcke@ipb-halle.de
